# Neuromodulation Reduces Interindividual Variability of Neuronal Output

**DOI:** 10.1523/ENEURO.0166-22.2022

**Published:** 2022-08-05

**Authors:** Anna C. Schneider, Omar Itani, Dirk Bucher, Farzan Nadim

**Affiliations:** Federated Department of Biological Sciences, New Jersey Institute of Technology and Rutgers University, Newark, New Jersey 07102

**Keywords:** bursting neuron, central pattern generator, stomatogastric, variability

## Abstract

In similar states, neural circuits produce similar outputs across individuals despite substantial interindividual variability in neuronal ionic conductances and synapses. Circuit states are largely shaped by neuromodulators that tune ionic conductances. It is therefore possible that, in addition to producing flexible circuit output, neuromodulators also contribute to output similarity despite varying ion channel expression. We studied whether neuromodulation at saturating concentrations can increase the output similarity of a single identified neuron across individual animals. Using the lateral pyloric (LP) neuron of the crab stomatogastric ganglion, we compared the variability of *f–I* (frequency–current) curves and rebound properties in the presence of neuropeptides. The two neuropeptides we used converge to activate the same target current, which increases neuronal excitability. Output variability was lower in the presence of the neuropeptides, regardless of whether the neuropeptides significantly changed the mean of the corresponding parameter or not. However, the addition of the second neuropeptide did not add further to the reduction of variability. With a family of computational LP-like models, we explored how increased excitability and target variability contribute to output similarity and found two mechanisms: saturation of the responses and a differential increase in baseline activity. Saturation alone can reduce the interindividual variability only if the population shares a similar ceiling for the responses. In contrast, the reduction of variability due to the increase in baseline activity is independent of ceiling effects.

## Visual Abstract

**Figure:**
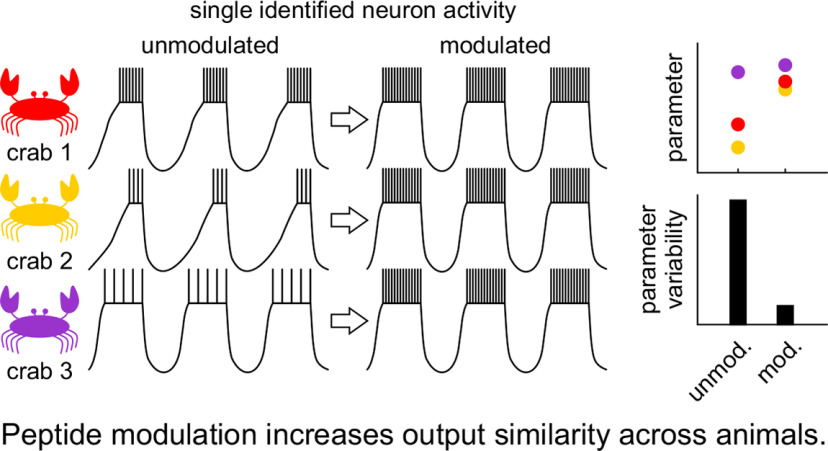

## Significance Statement

The activity of single neurons and neural circuits can be very similar across individuals although the ionic currents underlying activity are variable. The mechanisms that compensate for the underlying variability and promote output similarity are poorly understood but may involve neuromodulation. Using an identified neuron, we show that neuropeptide modulation of excitability can reduce interindividual variability of response properties at a single-neuron level in two ways. First, the neuropeptide increases baseline excitability in a differential manner, resulting in similar response thresholds. Second, the neuropeptide increases excitability toward a shared saturation level, promoting similar maximal firing rates across individuals. Such tuning of neuronal excitability could be an important mechanism compensating for interindividual variability of ion channel expression.

## Introduction

Under similar behavioral conditions across individual animals, neural circuits often produce very similar outputs. Output similarity is observed in all parts of the central nervous system, from spinal cord circuits (Pearson and Rossignol, 1991; Masino and Fetcho, 2005) to large networks involved in learning and memory (Wang, 2010; Howe et al., 2011; Buzsáki and Wang, 2012). Interestingly, interindividual similarity of circuit output is also present in the absence of sensory feedback, as has been observed in isolated invertebrate neural circuits (Hughes and Wiersma, 1960; Selverston and Moulins, 1985; Harris-Warrick and Marder, 1991; Büschges et al., 1995; Marder and Bucher, 2001; Bucher et al., 2005; Mulloney et al., 2006; Wenning et al., 2018). Such similarity is remarkable because, in any neuron type, ionic conductances underlying activity are quite variable. Even in a neuron that exists in only a single copy in each animal, as is common in small invertebrate circuits, the expression of ion channel conductances and their corresponding mRNA levels can vary several-fold, yet the activity of that neuron in the circuit remains remarkably similar (Prinz et al., 2004; Schulz et al., 2006; Goaillard et al., 2009; Tobin et al., 2009; Ransdell et al., 2013a; Marder et al., 2015; Tran et al., 2019). This observation raises the question of how neurons and circuits produce similar outputs despite variable components. A possible explanation is that different voltage-gated conductances are coregulated in a compensatory manner to give rise to similar neuronal excitability across individuals (MacLean et al., 2003, 2005; Khorkova and Golowasch, 2007; Harris-Warrick and Johnson, 2010; Temporal et al., 2012). Alternatively, output similarity may be an emergent property of the full circuit, as compensation for intrinsic variability may include the variability of synaptic strengths or constraints on synaptic current trajectories (Prinz et al., 2004; Anwar et al., 2022).

Output similarity is observed across individuals in comparable behavioral or circuit states, but circuit output is flexible and can change substantially depending on the behavioral needs of the animal. One mechanism providing such flexibility is neuromodulation by a variety of transmitters and hormones that modify properties of ion channels, synaptic release mechanisms, and other circuit components (Marder and Weimann, 1992; Katz et al., 1994; Parker, 2000; Johnson et al., 2011; Bargmann, 2012; Marder, 2012; Nadim and Bucher, 2014). This poses an interesting problem for output similarity. Neuromodulator receptor expression itself can show substantial interindividual variability (Garcia et al., 2015), suggesting that, in addition to the variability of expression, ionic conductances are also subject to variability of neuromodulator effects.

In the pyloric circuit of the stomatogastric ganglion (STG), conductance variability is substantial in both unmodulated and modulated states (Schulz et al., 2006; Khorkova and Golowasch, 2007; Golowasch, 2014; Anwar et al., 2022), indicating that receptor variability does not simply compensate for target ion channel variability. However, neuromodulatory effects interact with the variability of intrinsic neuronal properties, which may lead to reduction in output variability. In addition, output similarity may increase if a modulator increases neuronal excitability toward a saturation level that is shared across individuals. Voltage-gated ionic currents are nonlinear and, in any neuron type, interact in a complex but specific manner to produce neuronal excitability (Goaillard et al., 2009). It is therefore possible that, despite their varying levels, ionic currents interact in a manner that produces consistent output across individuals. The flexibility provided by neuromodulation may fine-tune these interactions to enhance neuronal output similarity. Finally, circuits are under the influence of several neuromodulators at all times, and while some neuromodulators have divergent cellular effects, others converge to modify the properties of some of the same ion channels or synapses (Bucher and Marder, 2013; Nadim and Bucher, 2014). Convergent comodulation may contribute to interindividual output similarity.

We focused on whether neuromodulation can increase output similarity at the level of an isolated neuron and, if so, whether comodulation by convergent neuromodulators enhances this similarity. We addressed these questions in the lateral pyloric (LP) neuron of the STG, which exists as a single copy in each animal. We first examined whether modulator-activated current levels in the LP neuron show similar levels of variability as other ionic currents. We then measured excitability, postinhibitory rebound properties, and response to periodic input in the synaptically isolated LP neuron. Subsequently, we compared the interindividual variability of these measures of activity in control conditions and in the presence of one or two (convergent) peptide neuromodulators. We used the experimental data to construct families of computational LP model neurons, with the same variability range as the data, to understand the mechanisms through which neuromodulation can influence interindividual variability at the single-cell level.

## Materials and Methods

### Experimental preparation

All experiments were performed on adult male Jonah crabs, *Cancer borealis*. Animals were obtained from local seafood stores in Newark, NJ, and kept unfed in tanks at 10–13°C. Crabs were placed in ice for at least 30 min for anesthetization before dissections. The stomatogastric nervous system (STNS) was dissected and pinned dorsal side up in a Sylgard (Ellsworth Adhesives) lined Petri dish. The dorsal sheath of the STG was removed with fine tungsten pins. During experiments, the STG was constantly perfused in a petroleum jelly well with 10–13°C saline at a flow rate of ∼4 ml/min. *C. borealis* saline contained the following (in mm): 440 NaCl, 26 MgCl_2_, 13 CaCl_2_, 11 KCl, 10 Tris base, and 5 maleic acid, buffered to pH 7.4.

The majority of chemical synapses in the pyloric circuit are graded, inhibitory, and glutamatergic, and can be blocked with picrotoxin (PTX). PTX (Sigma-Aldrich) was dissolved in DMSO (Thermo Fisher Scientific) and stored as a 10^−2^ m stock solution at 4°C. The PTX stock solution was diluted in saline to a final concentration of 10^−5^ m immediately before use. During voltage-clamp (VC) experiments, neurons were prevented from spiking by blocking sodium channels with tetrodotoxin (TTX). TTX (Alomone Labs) was dissolved in distilled water as a 10^−4^ m stock solution and stored at 4°C. The TTX stock solution was diluted in saline to a final concentration of 10^−7^ m immediately before use. Proctolin (Proc) and crustacean cardioactive peptide (CCAP; both custom synthesized by RS Synthesis) were dissolved individually in distilled water and stored as 10^−3^ m aliquots at −20°C. The neuropeptide stock solutions were diluted in TTX or PTX saline immediately before use to a final concentration of 10^−6^ M Proc and 5 × 10^−7^ m Proc + 5 × 10^−7^ m CCAP (henceforth referred to as Proc + CCAP) so that the total neuromodulator concentration in the single-modulator and double-modulator saline was always the same. All chemicals were always bath-applied to the STG.

#### Electrophysiology

The pyloric rhythm was recorded extracellularly with stainless steel pin electrodes inserted into petroleum jelly wells around sections of the lateral ventricular nerve (lvn), pyloric dilator nerve (pdn), and pyloric nerve. Extracellular electrodes were connected to a differential AC amplifier (model 1700, A-M Systems). The LP and pyloric dilator (PD) neurons were identified by matching their intracellular recorded activity to the extracellularly recorded pyloric rhythm.

We used two-electrode VC to measure synaptic and voltage-gated currents, and current clamp (CC) to measure excitability. Intracellular electrodes were made from thin-walled borosilicate capillaries with filaments, pulled to a sharp tip using a Flaming-Brown P-97 puller (Sutter Instrument), and filled with 0.6 m K_2_SO_4_ + 20 mm KCl (resistance: 20–25 MΩ). Intracellular signals were amplified using Axoclamp 900A amplifiers (Molecular Devices). All recordings were digitized at 5 kHz (Digidata 1440A, Molecular Devices) and recorded with Clampex 10.6 (Molecular Devices).

**Figure 1.**
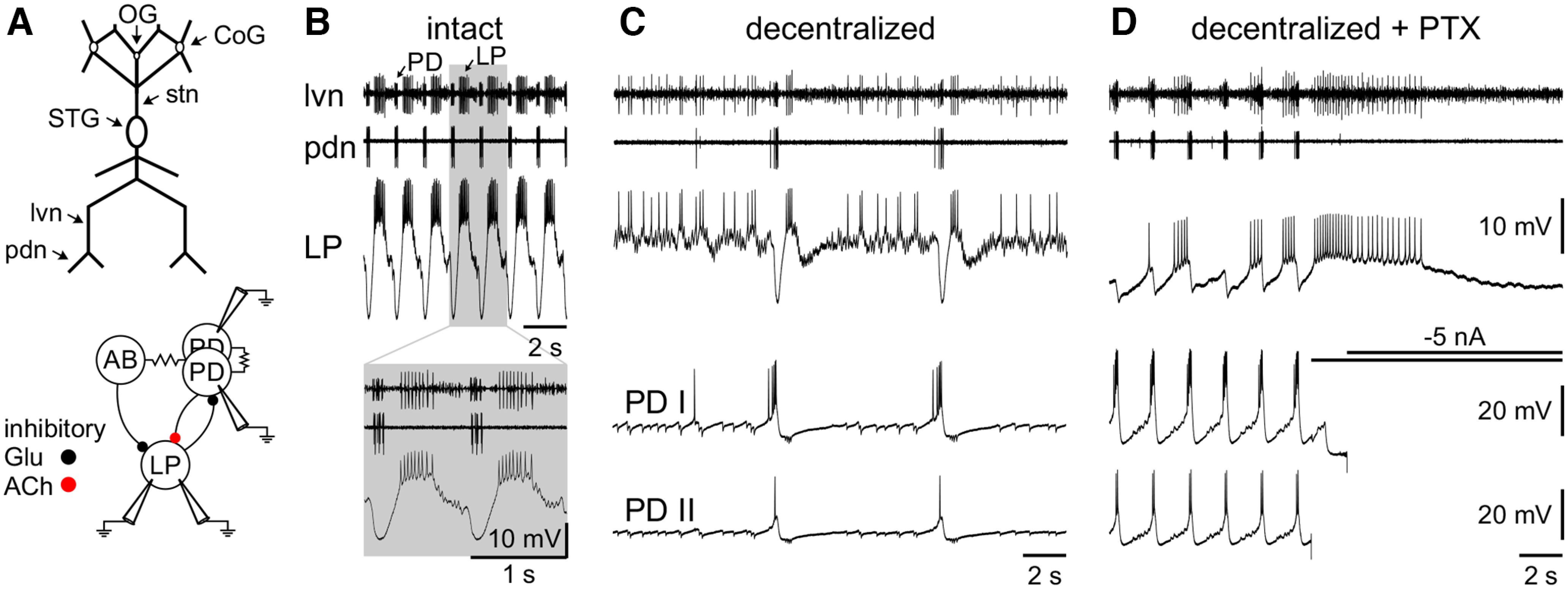
Isolating the LP neuron. ***A***, Schematic of the STNS (top) and the simplified pyloric network with electrodes to indicate which neurons were recorded from (bottom). Modulatory projection neurons are located in the commissural ganglia (CoG) and esophageal ganglion (OG) and project via the stn to the neurons in the STG. Neurons of the pyloric network are located in the STG. The network consists of a pacemaker group (one AB and two PD neurons), and several follower neurons, of which only the LP neuron provides direct chemical feedback to the pacemaker group. Chemical inhibitory synapses and their transmitters (glutamate or acetylcholine) are shown as circles, electrical coupling is depicted with resistor symbols. ***B–D***, Extracellular recordings of the lvn and pdn, which carry axons of both LP (largest unit on lvn) and PD neurons (mid-sized units on lvn), or only of PD neurons, respectively, and intracellular recordings of LP and the two copies of PD. ***B***, When the preparation is intact (all intrinsic neuromodulators present), the LP neuron receives strong periodic inhibition from the pacemaker group. ***C***, After decentralization (removal of intrinsic neuromodulators by transecting the stn), the pyloric rhythm deteriorates but synaptic connections are still functional as illustrated by LP inhibition during PD bursts, and IPSPs in PD for each LP spike. ***D***, After the addition of 10^−5^ m PTX, glutamatergic synapses between the pacemaker group and LP are blocked. The cholinergic synapse from PD to LP is still functional. When PDs are hyperpolarized (traces clipped), this synapse is silenced. Recordings in all three panels are from the same experiment and, with exception of the inset in ***B***, on the same voltage scale.

After cell identification, we removed all neuromodulatory inputs by transecting the stomatogastric nerve (stn; decentralization; Fig. 1). Unless otherwise indicated, we ran each of the protocols in decentralized control, Proc saline, Proc + CCAP saline, and after washing out. We did not wash between single and dual neuromodulator applications. Durations for wash in were 10 min and for wash out were 10–30 min before running a set of VC or CC protocols. In five experiments, we used sham wash in/wash out without any modulators in the saline to control for potential rundown of the LP neuron over time. Before and after each set of protocols, we measured the input resistance of the LP neuron by injecting currents pulses of −1 nA for 500 ms (10 sweeps). Experiments were discarded if input resistance was <5 MΩ.

#### Measurement of ionic currents

**Figure 2.**
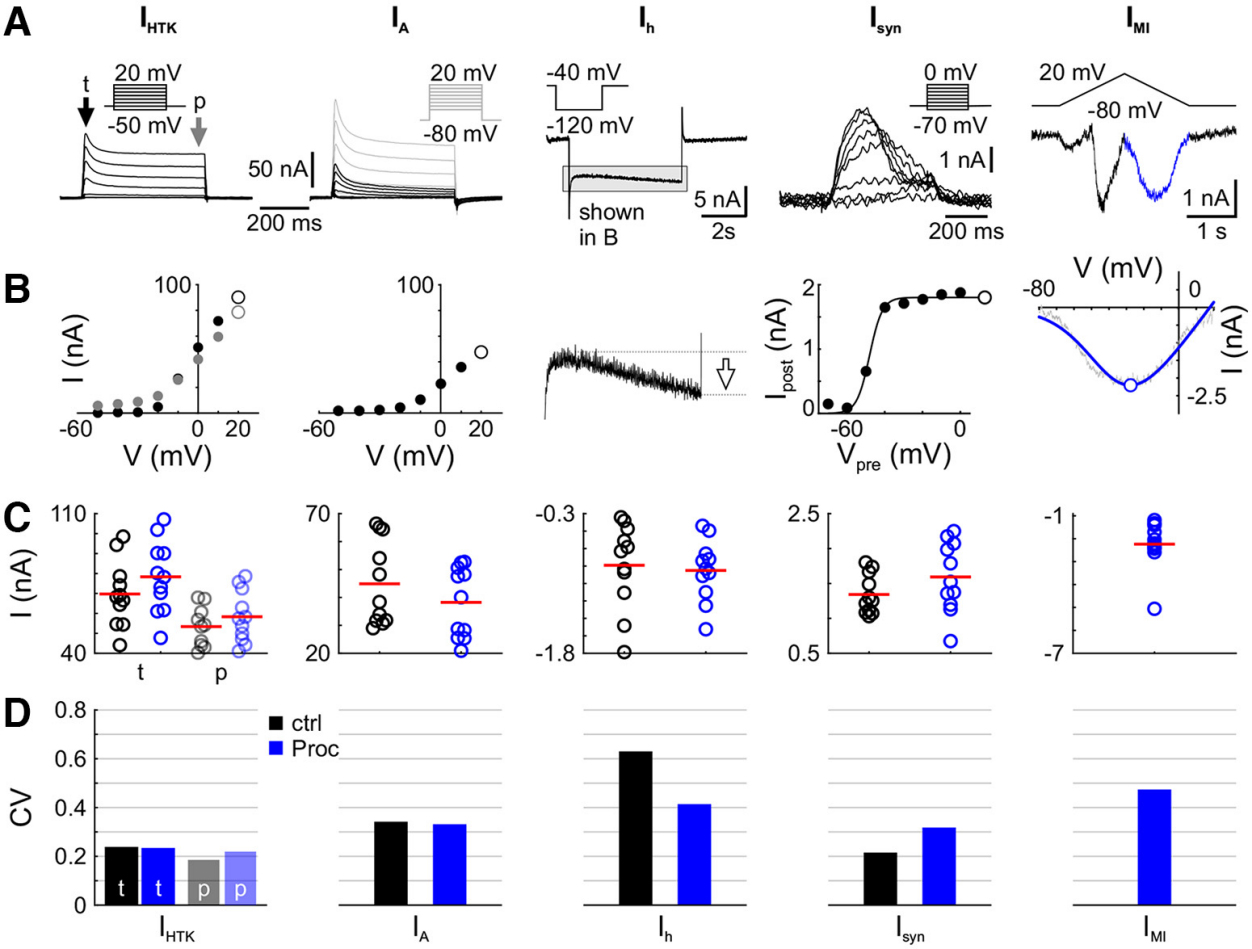
Variability of modulated components is not different from nonmodulated components across individuals. ***A***, Example voltage-clamp recordings for nonmodulated and modulated currents. Current traces for *I*_HTK_ [transient (t) portion indicated by black arrow, persistent (p) by gray arrow], *I*_A_ (with total potassium currents *I*_K_ as gray overlay; *I*_A_ is the difference current between all potassium currents *I*_K_ and *I*_HTK_); *I*_h_; the synaptic current *I*_syn_ from PD to LP; and the neuromodulator-activated, voltage-gated current *I*_MI_ are shown with the corresponding voltage-clamp protocol as the inset. ***B***, From these voltage traces, the features indicated with white markers were extracted: the maximum transient current for both potassium currents and additional the persistent current for *I*_HTK_, the amplitude of the current at the end of the hyperpolarizing voltage step for *I*_h_; the scaling factor for the sigmoid fit for *I*_syn_; and the maximum inward current of the current fit for *I*_MI_. ***C***, The distribution of these parameters across experiments are shown. Each dot represents one experiment, and red lines mark the mean. Black is for control (decentralized), blue is for Proc. Transient *I*_HTK_ is shown in bold colors, persistent in transparent colors. Since *I*_MI_ is calculated as a difference current (Proc – ctrl), there are no data in control. ***D***, Coefficients of variation (SD/mean) are in the same range for the nonmodulated currents (*I*_HTK_, *I*_A_, *I*_h_) and modulated currents (*I*_syn_, *I*_MI_).

For VC experiments, we applied 10^−7^ m TTX to block transient sodium currents, and then measured several ionic currents in the LP neuron in control and Proc saline. These currents included delayed rectifier and calcium-activated potassium currents measured together as the high-threshold potassium current (*I*_HTK_; Khorkova and Golowasch, 2007), the transient potassium current (*I*_A_), the hyperpolarization-activated inward current (*I*_h_), the synaptic current from the PD to LP synapse (*I*_syn_), and the modulator-activated inward current (*I*_MI_; Golowasch and Marder, 1992b; Fig. 2*A*). For these measurements, the PD neuron was always held at −60 mV, except during the *I*_syn_ measurements. For the total potassium currents (*I*_K_), the LP neuron was held at −80 mV and stepped for 500 ms from −50 to 20 mV in 10 mV increments. *I*_HTK_ was measured in the same way, except that LP was held at −40 mV to inactivate *I*_A_. *I*_A_ was calculated as the difference current between *I*_K_ and *I*_HTK_. To measure *I*_h_, we stepped LP from −40 to −120 mV for 10 s. For *I*_syn_, the LP neuron was held at −50 mV and the presynaptic PD neuron was stepped from −70 to 0 mV in 10 mV increments and a 500 ms step duration. To measure the noninactivating *I*_MI_, the voltage of the LP neuron was ramped from −80 to 20 mV (75 mV/s) and back to −80 mV. The *I–V* curve was then calculated as the difference between the current responses to the negative ramp in Proc and control saline (Schneider et al., 2021).

We used the maximum current level of each current in each experiment to calculate the variability of that component. For potassium currents, we used the value in response to the voltage step to +20 mV (Fig. 2*B*, open circles) for all further calculations. *I*_A_ was calculated as the difference in current between *I*_K_ (Fig. 2*A*, gray traces) and *I*_HTK_. For *I*_HTK_, we measured the transient and persistent currents separately, indicated by the arrows “t” and “p” in Figure 2*A*. For *I*_h_, we calculated the current amplitude between the beginning of the hyperpolarizing step and the end, indicated by the arrow in Figure 2*B*. For *I*_syn_, we first fitted the *I–V* data from the presynaptic voltage and average postsynaptic current during each presynaptic voltage step with a logistic sigmoid function, as follows:

(1) $$f(V)=\frac{a}{1+\exp\frac{V-V_{1/2}}{k}},$$

where *a* is the maximum postsynaptic current, *V*_1/2_ is the voltage at midpoint, and *k* is the slope factor at *V*_1/2_. Lower bounds were set at −10 nA for *a* and −80 mV for *V*_1/2_. We used the value of *a* as the maximum synaptic current, indicated by the open circle in Figure 2*B*.

For *I*_MI_ , we first calculated the difference current between Proc and control. From this, we separated the *I–V* curves for the positive and negative voltage ramp because the positive ramp activates an additional inactivating component, *I*_MI-T_ (Schneider et al., 2021). The *I–V* curves of the negative ramps were fitted with the following function:

(2) $$f(V)=\frac{a(V-b)}{1+\exp\frac{V-V_{1/2}}{k}},$$

with the following bounds: 0 ≤ *a*, 0 ≤ *b* (mV) ≤ 40, −40 ≤ *V*_1/2_ (mV) ≤0, and 0.1 ≤ *k* (mV) ≤ 20. The maximum current level for *I*_MI_ was the minimum negative current obtained from the fit function, indicated by the open circle in Figure 2*B*.

The parameters we used (Fig. 2*B*, white markers) were as follows: for the potassium currents, the maximum current value at the voltage step to 20 mV; for *I*_h_, the maximum amplitude during the step to −120 mV; for *I*_syn_, the scaling factor of the sigmoid fit of the mean postsynaptic current at each voltage step; and for *I*_MI_, the maximum inward current of the fitted current. Since *I*_MI_ is activated only in the presence of neuropeptides and is calculated as a difference current, we are unable to provide control data for this current.

#### Measurements of neuronal excitability

To measure the variability of excitability, we chemically isolated the LP neuron with 10^−5^ m PTX before running the stimulation protocols, which blocks inhibitory glutamatergic synapses from the pacemaker anterior burster (AB) and follower PY (pyloric) neurons to the LP neuron, as well as all synapses from the LP neuron to its targets (Bidaut, 1980). After 20 min of wash in, when IPSPs from LP to PD were effectively blocked, one or both PD neurons were hyperpolarized by −5 nA current injection or voltage clamped at a holding potential of −90 mV to silence the cholinergic synapse from PD to LP (Martinez et al., 2019; Fig. 1*A*,*D*).

To measure the excitability of the LP neuron in the form of frequency*–*current (*f–I*) curves, we injected increasing current levels from 0 to 5 nA in 0.5 nA increments, with 5 s duration, followed by the reversed protocol with decreasing current levels from 5 to 0 nA to check for hysteresis.

We measured the rebound properties of the LP neuron in two ways. To examine rebound properties following a long hyperpolarization period, we hyperpolarized the LP neuron with a −5 nA DC current for 10 s, followed by a 10 s interval of no current injection. This protocol was repeated for five sweeps and allowed us to measure the complete histogram of the burst structure of the LP neuron on rebound from hyperpolarization. To measure the steady-state rebound properties of the LP neuron as experienced during normal pyloric activity, we periodically hyperpolarized the LP neuron with 20 current pulses, −5 nA, 1 s on and 1 s off. We empirically determined that this 0.5 Hz periodic stimulation reliably resulted in rebound spiking in all modulatory conditions. Higher-frequency stimulations did not reliably produce spiking at steady state in the LP neuron in control or wash and were therefore not analyzed.

**Figure 3.**
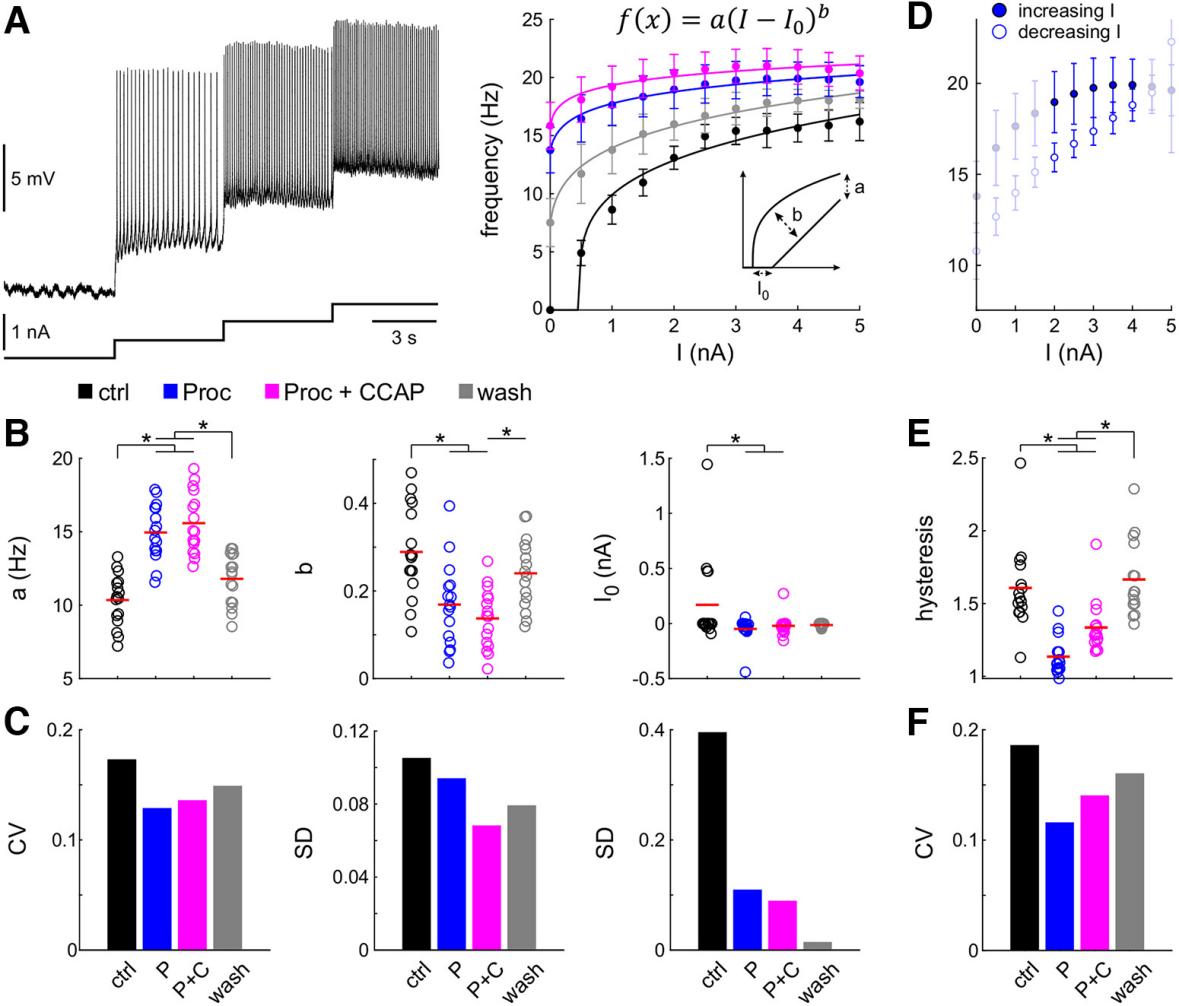
Neuromodulation changes excitability and reduces interindividual variability. ***A***, Example of f – I relationships in four modulatory conditions (black: control; blue: Proc; magenta: Proc + CCAP; gray: wash). Left panel: Intracellular recording at four levels of increasing current injection in control condition. Right panel: Instantaneous spike frequencies at each current level in different neuromodulatory states, shown as the average with SD, were fitted with a power function. The left inset shows how the fit parameters influence the appearance of the curve. ***B***, Distribution of fit parameters. Each dot represents the value from one experiment, and red lines indicate means. Application of one (blue, Proc) or two (magenta, Proc + CCAP) neuromodulators significantly changed parameters (asterisks). ***C***, Application of one or two neuromodulators reduced the variability of the fit parameters compared with control (black). ***D***, f – I curves showed hysteresis depending on in creasing (filled circles) or decreasing (open circles) levels of current injection. Only frequencies between 2 and 4 nA current injection (bold symbols) were used to calculate hysteresis as the ratio between increasing and decreasing current levels. ***E***, Distribution of hysteresis across experiments. Application of one or two neuromodulators significantly changed hysteresis (asterisks). ***F***, Application of one or two neuromodulators reduced the variability of hysteresis compared with control (black). Removing the outlier in control (i.e., the maximum value), for both hysteresis and *I*_0_, did not change the statistical significances. Control data for this figure are shown in Extended Data Figure 3-1. Raw data for B and E are provided in Extended Data Figure 3-2.

To compare the parameters of the *f–I* curves, we calculated the average instantaneous spike frequencies at each level of current injection and fit the experimental measurements with the following power function:

(3) $$f(I)=a{\left(I-I_{0}\right)}^{b},$$

where *a* is a scaling factor, *I*_0_ is the current level that first elicited spikes, and the power *b* was set to 0 ≤ *b *≤* *1 to limit the *f–I* curve to a sublinear function (Fig. 3*Ai*). To measure hysteresis of the *f–I* curve between depolarizing and repolarizing current injections (Fig. 3*Aii*), we calculated the average instantaneous spike frequency in the range of 2–4 nA and divided that value for the depolarizing current injections by that of the repolarizing current injections. This converted the hysteresis data to a ratio scale to use the coefficient of variation (CV) as a measure for variability. Ratios >1 mean that spike frequencies were greater during the current injections with depolarizing increments.

10.1523/ENEURO.0166-22.2022.f3-1 Figure 3-1 *f–I* relationships with sham applications of neuromodulators. One example experiment. ***A***, Increasing current application (indicated by arrows). ***B***, Decreasing current application (indicated by arrows). Download Figure 3-1, TIF file.

10.1523/ENEURO.0166-22.2022.f3-2 Figure 3-2 Raw data shown in Figure 3, *B* and *E*. Download Figure 3-2, XLS file.

**Figure 4.**
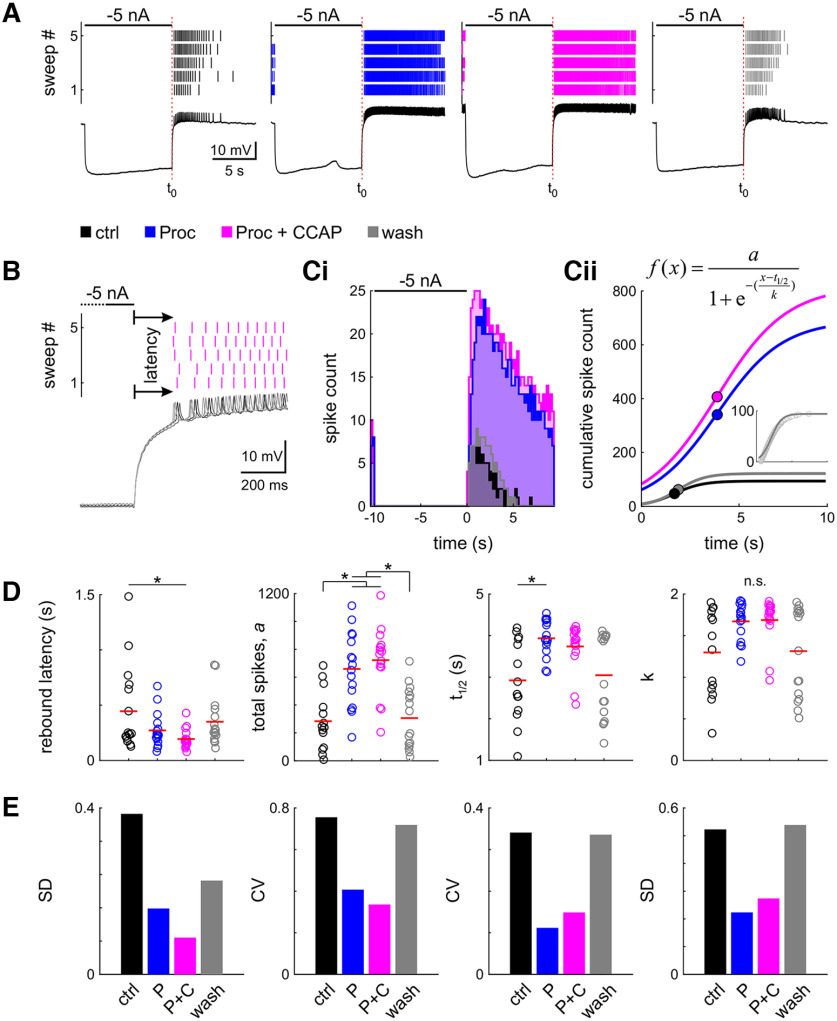
Neuromodulation changes general rebound properties and reduces interindividual variability. ***A***, Spike raster and intracellular recording of the last of the five sweeps of one example experiment in four modulatory conditions (black: control; blue: Proc; magenta: Proc + CCAP; gray: wash). ***B***, Latency was measured as the time from the end of the hyperpolarizing current injection to the first spike, averaged across all five sweeps. Sweeps of the intracellular recording are shaded in gray, from dark to light for sweeps 1–5. ***C***, Spike histogram (***Ci***, 200 ms bin size) and sigmoid fit to the cumulative spike histogram (***Cii***). Dots indicate sigmoid midpoint. ***A–C*** are from the same experiment. ***D***, Parameter distribution for latency and fit parameters; *t*_1/2_ is relative to the end of the current injection (***A***, *t*_0_). Dots represent individual experiments; the red line indicates the mean. Application of neuromodulators significantly (asterisks) changes most parameters (n.s.: ANOVA not significant). ***E***, Variability of all parameters is reduced in the presence of neuromodulators. Control data for this figure are shown in Extended Data Figure 4-1. Raw data for ***D*** are provided in Extended Data Figure 4-2.

**Figure 5.**
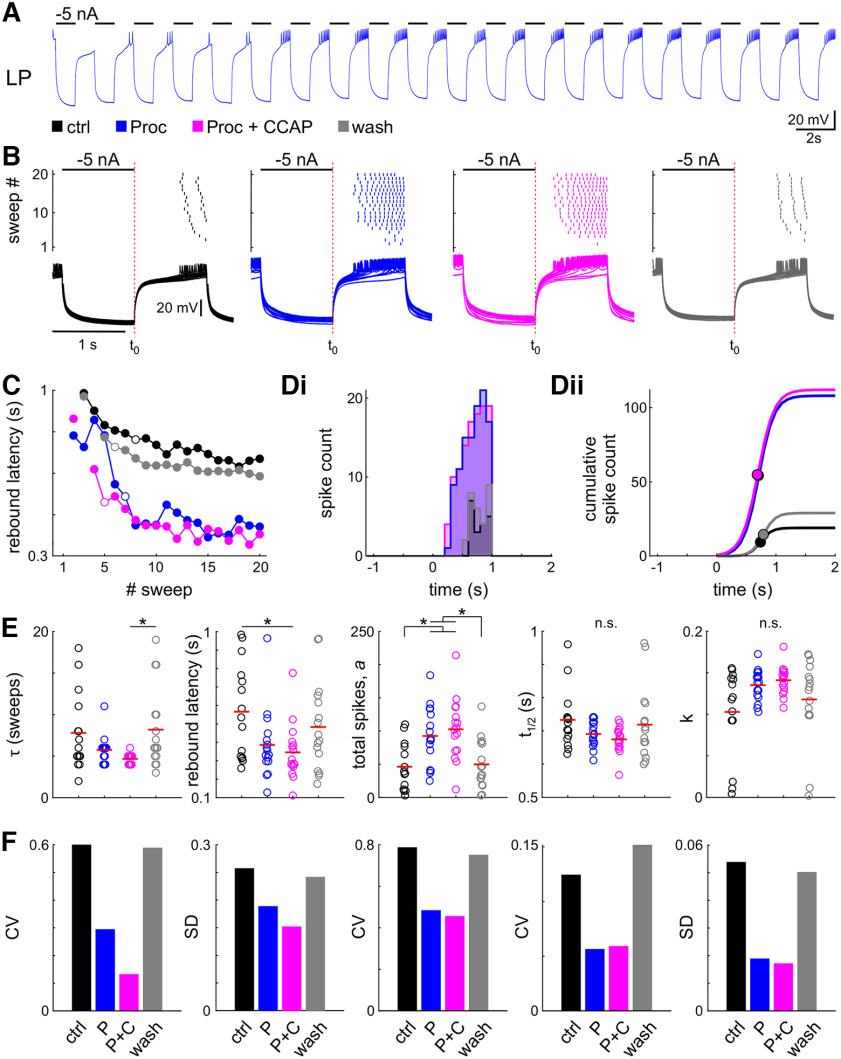
Neuromodulation changes steady-state rebound properties and reduces interindividual variability. ***A***, Example intracellular recording of the LP neuron with 20 cycles of periodic inhibition. ***B***, Spike raster and intracellular recording of all 20 sweeps of the same experiment in all four modulatory conditions (black: control; blue: Proc; magenta: Proc + CCAP; gray: wash). ***C***, During the first sweeps the latency to the first spike successively decreased; therefore, we only included the last 10 sweeps (steady state) in the further analysis. The open circles indicate the sweep at which the latency was reduced to 63% of its total range. ***D***, Spike histogram (***Di***, 100 ms bin size) and sigmoid fit to the cumulative spike histogram (***Dii***). Dots indicate sigmoid midpoint. ***A–D*** are from the same experiment. ***E***, Parameter distribution for latency and fit parameters; *t*_1/2_ is relative to the end of current injection (***B***, *t*_0_). Dots represent individual experiments; the red line indicates the mean. Application of neuromodulators significantly changes parameters. ***F***, Variability of all parameters is reduced in the presence of neuromodulators. Control data for this figure are shown in Extended Data Figure 5-1. Raw data for ***E*** are provided in Extended Data Figure 5-2.

To analyze rebound properties following a long hyperpolarizing DC current (Fig. 4), we considered all five sweeps because we did not observe transient effects across subsequent sweeps. For the rebound in response to periodic hyperpolarizing pulses (Fig. 5), we only considered the last 10 of the 20 pulses where the response of the LP neuron had reached steady state. In both cases, we calculated the latency to the first spike and the rebound spike structure for the selected sweeps (or pulses) in relation to the end of the hyperpolarizing current step. For the spike structure, we fitted the cumulative spike count histogram with the sigmoid function in Equation 1, in which *a* is now the maximum number of spikes per sweep or pulse and *t*_1/2_, instead of *V*_1/2_ , is the time of the midpoint relative to the end of the current injection. Additionally, for the steady-state rebound, we approximated the time constant by identifying the pulse number at which the latency dropped to 63% of its total drop value.

10.1523/ENEURO.0166-22.2022.f4-1 Figure 4-1 Rebound with sham applications of neuromodulators. One example experiment. ***A***, Spike raster and corresponding intracellular recordings for all five sweeps. ***B***, Spike histogram and sigmoid fit to the cumulative spike histogram. Dots indicate sigmoid midpoint. Download Figure 4-1, TIF file.

10.1523/ENEURO.0166-22.2022.f4-2 Figure 4-2 Raw data shown in Figure 4*D*. Download Figure 4-2, XLS file.

10.1523/ENEURO.0166-22.2022.f5-1 Figure 5-1 Periodic rebound with sham applications of neuromodulators. One example experiment. ***A***, Spike raster and corresponding intracellular recordings for all 20 sweeps. ***B***, Spike histogram and sigmoid fit to the cumulative spike histogram. Dots indicate sigmoid midpoint. Download Figure 5-1, TIF file.

10.1523/ENEURO.0166-22.2022.f5-2 Figure 5-2 Raw data of the graph shown in Figure 5*E*. Download Figure 5-2, XLS file.

### Data analysis

All data were analyzed using custom scripts written in MATLAB (releases R2018a and R2020b; MathWorks). Clampex files were imported with abfload (version 1.4.0.0; https://www.mathworks.com/matlabcentral/fileexchange/6190-abfload).

Statistical tests were performed with SigmaPlot (version 12.0; SyStat Software) or custom-written MATLAB scripts. Significance was assumed at α = 0.05. We performed one-way ANOVA when data passed the Shapiro–Wilk normality test and Levene’s equal variance test, or one-way ANOVA on ranks if at least one of the tests failed. We used Tukey’s test for *post hoc* multiple comparisons if the data had equal variance, and Dunn’s test if the variance was not equal. ANOVA results are listed in Tables 3, 4, and 5. We did not use paired tests because four of our single-cell variability experiments did not spike in control condition and were replaced with the control condition of four of the five sham experiments in which we never applied any modulators. Variability was calculated as CV (SD normalized to the absolute mean) and adjusted for the sample size (Haldane, 1955) if data were on a ratio scale, or as SD if data were on an interval scale. One exception is latency, where we also use SD as a variability metric. To calculate CV for latency, we must use the onset of the stimulus as a reference time point. Since we have stimulus durations of 10 s for most rebound experiments, the CVs in this case would be a scaled version of the SD.

The data presented are from 11 experiments for component (ionic current) variability, 16 experiments for single-cell (neuronal) variability, and 5 experiments with no modulator application.

#### LP model structure and activity

The LP neuron model was built as described in the study by Schneider et al. (2021), and implemented and run using NEURON (Carnevale and Hines, 2006) in Python. In brief, the model is composed of two coupled compartments, one representing the soma/neurite, and the other representing the axon. Spiking is generated in the axon, which has *I*_leak_, *I*_Na_, and *I*_K_, while the soma produced bursting activity with *I*_leak_, *I*_Ca_, *I*_KCa_, *I*_A_, *I*_h_, and *I*_MI_. Calcium accumulation was tracked, where *I*_Ca_ contributed to intracellular calcium concentration ([Ca]_in_), and [Ca]_in_ influenced both *I*_Ca_ and *I*_KCa_ as described in Table 1. In addition to *I*_Ca_, all currents were modeled as standard Hodgkin–Huxley type currents with the following general form:

(4) $$I_{x}={\bar{g}}_{x}m_{x}^{p}h_{x}^{q}(V-E_{x}),$$

(5) $$\frac{dz}{dt}=\frac{z_{\infty }(V)-z}{\tau_{z}(V)},$$

where *x* is the current type, ${\bar{g}}_{x}$ is the maximal conductance, *p* and *q* are, respectively, the activation (*m*) and inactivation (*h*) variable (non-negative integer) exponents, and *E_x_* is the reversal potential. Reversal potentials of axonal currents are *E*_leak_ = −55 mV, *E*_Na_ = 70 mV, and *E*_K_ = −80 mV. The reversal potentials of somatic currents are *E*_leak_ = −50 mV, *E*_KCa_ = −80 mV, *E*_A_ = −80 mV, *E*_h_ = −20 mV, and *E*_MI_ = −10 mV. The change of activation and inactivation terms is given by Equation 5, where *z_∞_* is the steady-state value of *m* or *h* and *τ*_z_ is the corresponding time constant. The parameters for the model are provided in Table 1. The calcium current was modeled using the Goldman–Hodgkin–Katz formalism, as follows:

(6) $$I_{\text{Ca}}=P_{\text{Ca}}m_{\text{Ca}}^{3}h_{\text{Ca}}F\zeta \left(\frac{{\left[Ca\right]}_{\text{out}}e^{-\zeta }-{\left[Ca\right]}_{\text{in}}}{e^{-\zeta }-1}\right),$$

(7) $$\zeta =\frac{z_{\text{Ca}}\text{vol}\cdot F}{\text{RT}}V,$$

where *P*_Ca_ is the total permeability of the current, *m*_Ca_ and *h*_Ca_ are activation and inactivation variables given by Equation 5, vol is the volume of the microdomain influencing the current, *F* is Faraday’s constant, *R* is the universal gas constant, and *T* is temperature. [*Ca*] is the calcium concentration outside (out) and inside (in) the cell. The internal calcium concentration is given by the following:

(8) $$\frac{d{[\text{Ca]}}_{\text{in}}}{dt}=\frac{{\left[\text{Ca}\right]}_{\infty }-{[\text{Ca]}}_{\text{in}}}{\tau_{\text{Ca}}}-\frac{P_{1}}{z_{\text{Ca}}F\cdot \text{vol}\cdot P}I_{\text{Ca}},$$

where *I*_Ca_ denotes the calcium current, [Ca]_∞_ denotes the steady-state calcium concentration inside the cell, *P*_1_ is the maximal per cluster permeability of *I*_Ca_, and *P* is the total permeability over all clusters of interest. The parameters of *I*_Ca_ are *P*_1_ = 0.014 (cm · ms)^−1^, *T *=* *283.15 K, [*Ca*]_out_ = 13 mm, *P*_1_ = 1.1675 μm^3^/s, *p *=* *0.0467 (cm · ms)^−1^, vol* *=* *6.49 μm^3^, [Ca]_∞_ = 0.02 mm, and τ_Ca_ = 25 ms.

**Table 1:** Model parameters for the LP-like model

		*m*_∞_	τ_m_	*h*_∞_	τ_h_
Soma/Neurite					
*I*_A_	*m^2^h*	$\text{S}\left(\frac{v+10}{20}\right)$	2	$\text{S}\left(-\frac{v+60}{75}\right)$	50
*I*_Ca_	*m^2^h*	$\text{S}\left(\frac{v+45}{15}\right)$	$100+2500*\text{S}\left(\frac{v+40}{20}\right)$	$\text{S}\left(-\frac{{\left[\mathrm{Ca}\right]}_{i}}{5e-4}\right)$	$500+2500*\text{S}\left(-\frac{v+70}{5}\right)$
*I*_h_	*m*	$\text{S}\left(\frac{v+70}{3}\right)$	$2500+1500*\text{S}\left(\frac{v+60}{4}\right)$		
*I*_K(Ca)_	*mh*	$\frac{1}{\left(1+{\left(\frac{1.43e-3}{{\left[Ca\right]}_{i}}\right)}^{5}\right)*\text{S}\left(\frac{v+5.5}{8}\right)}$	$\begin{array}{l} 499-494*\\ \text{S}\left(v+\frac{54.6*\log\left(\frac{{\left[Ca\right]}_{i}}{1e-3}\right)}{10}\right) \end{array}$	$\text{S}{\left(-\frac{{\left[Ca\right]}_{i}}{0.03}\right)}^{1.25}$	25
*I*_MI_	*m*	$\text{S}\left(\frac{v+48}{5}\right)$	5		
Axon					
*I*_Na_	*m^3^h*	$\text{S}\left(\frac{v+18}{12.25}\right)$	1	$\text{S}\left(-\frac{v+28}{7.7}\right)$	2.5
*I_K_*	*m^4^*	$\text{S}\left(\frac{v+23}{5}\right)$	$2+7*\text{S}\left(-\frac{v+23}{5}\right)$		

*S*(*x*) denotes the logistic sigmoid function 1/(1 + exp(–*x*)). Time constants are in milliseconds.

The difference between the LP model used here compared with the model in Schneider et al. (2021) is that, here, the kinetics of *I*_Ca_, *I*_A_, and *I*_h_ were tuned to capture rebound firing, as seen in the biological data of this study. The protocol for rebound firing in the model is the same as in the biological experiments, where −5 nA is injected into the LP model soma for 10 s on and 10 s off. The *f–I* curves were generated by injecting 5 s current steps into the LP soma. The current step amplitudes ranged from −10 to 5.5 nA in increments of 0.5 nA. This larger range was chosen to better fit the *f–I* power function curve parameter *I*_0_. Cumulative spike histograms of model rebound experiments, and *f–I* power function curves of model *f–I* experiments were fit using scipy.optimize (Virtanen et al., 2020).

#### Generating a family of LP models

To address the questions of the effects of modulation on population variability, families of models were generated such that their activity summary statistics matched those of the experimental observations in the control condition (decentralized). This was done by generating a pool of candidate models using the simulation-based inference toolbox SBI (Gonçalves et al., 2020; Tejero-Cantero et al., 2020), and then selecting the final family of models according to criteria constrained by experimental results. For the rebound firing case, the summary statistics matched were the latency and power function coefficients (*a*, *t*_1/2_, *k*). The model parameters estimated were the somatic maximal conductances (*g*_leak_, ${\bar{g}}_{A}$, ${\bar{g}}_{h}$, ${\bar{g}}_{\mathrm{KCa}}$, ${\bar{g}}_{\mathrm{MI}}$) and calcium permeability (*P*_Ca_). The selection criteria imposed on the rebound firing population were that rebound latency must be >0.1 and <1.5 s. This resulted in a family of models (*n* = 198) whose summary statistics matched the experimentally measured firing rebound statistics in the decentralized control condition.

A similar approach was used to generate a family of LP models whose *f–I* curve fit parameters matched those of experimental data in the control (decentralized) condition (*n* = 85). In this case, we used the *f–I* power function fit parameters *a*, *b*, and *I*_0_ as summary statistics and estimated the same model parameters as in the rebound case, as well as of axonal maximal conductances (*g*_leak_, ${\bar{g}}_{\mathrm{Na}}$, and ${\bar{g}}_{K}$) and the inactivation time constant (*τ*_h_) for *I*_Na_. The selection criteria used were the exclusions of models that went into depolarization block in the range of additional ${\bar{g}}_{\mathrm{MI}}$ added, and models where the *f–I* power function fit parameter *a* was <6.

#### Population of linear integrate-and-fire models

To check whether our observations could be explained from first principles, we used a standard linear integrate-and-fire (LIF) model. The change in voltage with respect to time is given as follows:

(9) $$C_{m}\frac{dv}{dt}=I_{\text{app}}-g_{\text{leak}}(v-E_{\text{leak}})-g_{\text{MI}}(v-E_{\text{MI}}),$$

where a spike occurs when voltage exceeds *v*_th_, after which the voltage is immediately set to *v*_reset_.

*C*_m_ is the membrane capacitance, and *I*_app_ is the amount of DC current injected. The ionic currents included were *I*_leak_ and *I*_MI-L_, a linearized version of *I*_MI_. Here, *I*_leak_ is a standard leak current with reversal potential *E*_leak_ = −60 mV and conductance *g*_leak_, and *I*_MI-L_ is a positive leak current with reversal potential *E*_MI_ = 10 mV and conductance *g*_MI-L_. All values used are given in Table 2.

**Table 2:** Parameter values used for LIF simulations

Parameter	Value
*C_m_*	0.1 μF
*I*_app_	[−50:100] nA
*g*_leak_	Uniform distribution [0.05, 0.2) μS
*E*_leak_	−60 mV
*g*_MI-L_	0, 0.2, 0.36 μS
*E*_MI_	10 mV
*v*_th_	−40 mV
*v*_reset_	−80 mV

At steady state, the frequency of the LIF neuron is given as follows:

(10) $$f=\frac{1}{\tau_{\text{m}}\ln\frac{v_{\text{ss}}-v_{\text{reset}}}{v_{\text{ss}}-v_{\text{th}}}}.$$

The membrane time constant (τ_m_) is as follows:

(11) $$\tau_{\text{m}}=\frac{C_{\text{m}}}{g_{\text{leak}}+g_{\text{MI-L}}}.$$

Steady-state voltage (*v*_ss_) is as follows:

(12) $$v_{\text{ss}}=\frac{I_{\text{app}}+g_{\text{leak}}E_{\text{leak}}+g_{\text{MI-L}}E_{\text{MI}}}{g_{\text{leak}}+g_{\text{MI-L}}}.$$

A family of LIF model was constructed where *g*_leak_ was sampled from a uniform distribution while all other parameters were kept fixed. To increase excitability, we added fixed amounts of ${\bar{g}}_{\mathrm{MI-L}}$ to the family of LIF models. The frequencies of the models were calculated using Equation 10 and fit to Equation 3, and the measures of variance were calculated in the same way as for the biological data.

### Data availability

Source code for the LP model and linear integrate-and-fire model is available at https://github.com/fnadim/Schneider_et_al_variabilty_single_neuron and as Extended Data 1.

10.1523/ENEURO.0166-22.2022.ed1 Extended Data 1 Supplementary Model code. Download Extended Data 1, ZIP file.

## Results

### Interindividual variability is not different between nonmodulated and modulated ionic currents

Most neurons express a multitude of ionic currents and receive synaptic input from many neurons. A modulatory input often activates or modifies the levels of a subset of these ionic currents. Substantial interindividual variability has been shown in identified pyloric neurons both for unmodulated voltage-gated currents (Schulz et al., 2006; Hamood and Marder, 2014), modulator-activated currents (Goaillard et al., 2009), and synaptic currents (Goaillard et al., 2009; Anwar et al., 2022). As a first step, we wanted to confirm the substantial interindividual variability of ionic currents found in the LP neuron and that neuropeptide neuromodulation does not systematically change component variability. We compared the variability of ionic currents that are targeted by proctolin and currents that are not, by measuring these currents in the same preparations. The nonmodulated currents that we measured included *I*_HTK_ (transient and persistent portions), *I*_A_ and *I*_h_, and the modulated currents included *I*_syn_ and *I*_MI_ (Fig. 2*A*,*B*; Golowasch and Marder, 1992b; Li et al., 2018). All currents except *I*_MI_ were measured twice, once in control and once in Proc (Fig. 2*C*), whereas *I*_MI_ was measured as a difference current between control and Proc (see Materials and Methods). For each current, the CV (SD divided by the absolute mean) was calculated across individuals (Fig. 2*D*). As expected, CV values were larger for the currents that had a small overall magnitude (*I*_h_ and *I*_MI_), likely because of increased measurement error, and therefore larger SD, for small currents. Because CV is the ratio of SD to the mean, changes in CV can be because of the following three mechanisms: (1) if the mean is constant but the SD is increasing, CV would increase; (2) if SD is constant but the mean increases, CV would decrease; and (3) if both mean and SD are changing, changes in CV would depend on the ratio changes of both. Generally, these measurements showed that CV values were similar between modulated and nonmodulated currents, and between control and Proc. Hence, the addition of Proc did not systematically reduce current variability, and the variability of modulated currents is in the same range as that of nonmodulated currents. Consequently, any change in variability of neuronal response properties caused by neuropeptide modulation is not simply because of altered component variability, but must arise from the interactions of ionic currents.

### Neuromodulation reduces interindividual variability of the *f–I* relationship

The *f–I* relationship of a neuron describes the gain of its input–output function and is an established measure of neuronal excitability (Ermentrout, 1996; Skinner, 2013). We therefore measured the variability of the *f–I* relationship of the LP neuron across preparations (Fig. 3). We generated *f–I* curves by applying different levels of DC current to the LP neuron and measuring its firing frequency at each current level. To compare the *f–I* relationships across preparations, we used the *f–I* data from the increasing current steps and fit this curve with a power function for each modulatory condition (control, Proc, Proc + CCAP, wash; Fig. 3*A*). We fitted the *f–I* curves to obtain three parameters that capture the relationship and compared the fit parameters across all modulatory conditions (Fig. 3*B*).

Application of neuromodulators consistently altered the *f–I* curve of the LP neuron (Table 3, one-way ANOVA results). In contrast, sham neuromodulator application (see Materials and Methods) did not change the *f–I* relationship (Extended Data Fig. 3-1, example). Neuromodulators significantly increased the scaling factor *a*, indicating that the spike frequency of the LP neuron increased at any applied current level in the presence of neuromodulators. This is a well known phenomenon and is expected from modulators that activate inward currents (Heckmann et al., 2005). The exponent *b* decreased with modulators, indicating that the firing frequency started saturating at lower current levels, as higher values of *b* (closer to 1) indicate a more linear *f–I* relationship. Finally, modulator application lowered the firing threshold, as *I*_0_, the current level at which LP first spiked, decreased significantly, again an expected and well known phenomenon (Binder et al., 1993).

**Table 3:** One-way ANOVA results for *f–I* experiments

*N*	Parameter	Normality	Variance	Test statistic	df	res	*p*	ctrl	Proc	Proc + CCAP	Wash
16	a	pass	pass	*F* = 28.703	3	60	**<0.001**	10.346	14.947	15.583	11.790
16	b	pass	pass	*F* = 9.805	3	60	**<0.001**	0.289	0.167	0.137	0.240
16	I_0_	fail		*H* =11.289	3		**0.01**	0.000	−0.020	−0.026	−0.013
16	hysteresis	fail		*H* = 40.453	3		**<0.001**	1.554	1.092	0.128	1.581

“*N*” is the number of animals, results from tests for normality and equal variance is given as pass/fail, the test statistics are *F* for ANOVA and *H* for ANOVA on ranks, and “res” is the residuals. Significant *p*-values (*p* ≤ 0.05) are printed in bold. Values for ctrl, Proc, Proc + CCAP, and wash are means for ANOVA, and medians for ANOVA on ranks.

To compare interindividual variability of the parameters under different modulatory conditions, we calculated the CV for parameter *a*, and the SD for parameters *b* and *I*_0_ (Fig. 3*C*; see Materials and Methods). For all parameters, variability was highest in control and decreased with modulator application. After the wash, variability of all parameters except for *I*_0_ increased. For all parameters, variability was reduced in the presence of modulators. However, the coapplication of Proc and CCAP did not consistently decrease variability more than Proc on its own. Since the SD for *a* was similar among the four conditions, the decrease in CV was because of the increased mean in Proc and Proc + CCAP compared with control and wash.

The *f–I* relationship with decreasing current steps was not identical to that with increasing steps (Fig. 3*D*), indicating hysteresis (Lee and Heckman, 1998). To quantify this hysteresis, we used the average instantaneous frequencies for current step values between 2 and 4 nA and measured the ratio of this measurement for increasing over decreasing steps (Fig. 3*E*, Table 3). This ratio was >1 in all conditions meaning that, at the same current level, the LP neuron firing frequency was always larger when increasing the applied current than decreasing it. With modulators, the ratio was closer to 1, indicating a reduction in hysteresis. As for the *f–I* curve fit parameters, modulator application reduced the interindividual variability of hysteresis, as seen in the smaller CV values (Fig. 3*F*). The decrease in CV together with the decrease in the respective means indicated a strong contribution of the reduction of SD. Raw data for these calculations is available in Extended Data Figure 3-2.

### Neuromodulation reduces interindividual variability of rebound properties

In the intact pyloric network, the LP neuron generates bursts of spikes when it rebounds from inhibition by the pacemaker neurons. To assess rebound properties, we recorded the responses after release from a 10 s hyperpolarizing current step of −5 nA under different neuromodulatory conditions (Fig. 4*A*). We determined the latencies to the first rebound spike (Fig. 4*B*) and generated histograms of the rebound spike trains for the 10 s window after hyperpolarization (Fig. 4*Ci*). From the histograms, we fitted the cumulative spike counts with a sigmoid to extract parameters that can be used for comparisons of variability (Fig. 4*Cii*). Sham neuromodulator application (see Materials and Methods) did not change the rebound properties (Extended Data Fig. 4-1, example).

The LP neuron was usually silent in the absence of modulators. Upon hyperpolarization, we observed a slow voltage sag, which is known to be due to the activation of *I*_h_ (Fig. 4*A*; Golowasch and Marder, 1992a). Upon rebound, the LP neuron fired a train of spikes that terminated within the 10 s recording interval. In the presence of Proc or Proc + CCAP, however, LP was more depolarized and tonically firing without external current input. Upon hyperpolarization, it stopped spiking and produced a similar sag as in the control saline. In all conditions, the LP neuron produced a rebound after hyperpolarization and then its spike rate tapered off to its baseline level (Fig. 4*A*). The LP neuron produced its rebound spiking with a brief latency (Fig. 4*B*). In each condition, this latency to the first spike was similar for all sweeps. However, the application of Proc + CCAP significantly reduced the latency compared with control (Fig. 4*D*, Table 4). The spike histogram reflected that the spike rate first increased after the release from hyperpolarization and then tapered off (Fig. 4*Ci*). The total spike number, measured as the sigmoid fit parameter *a*, significantly increased in the presence of neuromodulators (Fig. 4*D*, Table 4), consistent with the increased excitability we observed in the *f–I* curves. The *t*_1/2_, relative to the release from hyperpolarization, occurred later with Proc than in control, but *k*, the steepness of the cumulative spike histogram, was not significantly changed with any of the modulators (Fig. 4*D*, Table 4). However, the variability of all parameters was always reduced in the presence of neuromodulators, indicated by lower values of SD and CV, regardless of any changes in the parameter (Fig. 4*E*). Variability of all parameters returned toward control levels in wash. For rebound latency, the decreased CV was because of a larger decrease in SD than the mean. For *a*, the SD increased, which was compensated for by the larger increase in the mean. For *t*_1/2_, the mean was increased and the SD was decreased, resulting in a smaller CV. Raw data for these calculations are available in Extended Data Figure 4-2.

**Table 4:** One-way ANOVA results for general rebound experiments

*N*	Parameter	Normality	Variance	Test statistic	df	res	*p*	ctrl	Proc	Proc + CCAP	Wash
16	Log latency	Pass	Pass	*F* = 3.675	3	60	**0.017**	−1.097	−1.444	−1.725	−1.208
16	*a*	Pass	Pass	*F* = 14.937	3	58	**<0.001**	283.857	659.188	722.063	306.063
16	*t*_1/2_	Pass	Fail	*H* = 10.337	3		**0.016**	2.741	3.901	3.899	3.349
16	*k*	Fail		*H* = 6.709	3		0.082	1.415	1.730	1.780	1.522

“*N*” is the number of animals, results from tests for normality and equal variance is given as pass/fail, the test statistics are *F* for ANOVA and *H* for ANOVA on ranks, and “res” is the residuals. Significant *p*-values (*p* ≤ 0.05) are printed in bold. Values for ctrl, Proc, Proc + CCAP, and wash are means for ANOVA, and medians for ANOVA on ranks. Latency was bounded by experimental design between 0 and 10; therefore, we logarithmically transformed the data to a normal distribution and did the statistics on the log-transformed data.

**Table 5:** One-way ANOVA results for steady-state rebound experiments

*N*	Parameter	Normality	Variance	Test statistic	df	res	*p*	ctrl	Proc	Proc + CCAP	Wash
16	τ	Fail		*H* = 10.310	3		**0.016**	6	6	5	6
16	arctan (latency*π)	Pass	Pass	*F* = 3.124	3	59	**0.033**	0.996	0.831	0.786	0.919
16	*a*	Pass	Pass	*F* = 7.623	3	58	**<0.001**	46.286	92.563	102.688	49.813
16	*t*_1/2_	Fail		*H* = 3.791	3		0.285	0.714	0.690	0.678	0.691
16	*k*	Fail		*H* = 5.115	3		0.164	0.119	0.138	0.144	0.131

“*N*” is the number of animals, results from tests for normality and equal variance are given as pass/fail, the test statistics are *F* for ANOVA and *H* for ANOVA on ranks, and “res” is the residuals. Significant *p*-values (*p* ≤ 0.05) are printed in bold. Values for ctrl, Proc, Proc + CCAP, and wash are means for ANOVA, and medians for ANOVA on ranks. Latency was bounded between 0 and 1 by experimental design. Therefore, we transformed the data to a normal distribution by multiplying by π and calculating the arctangent.

### Neuromodulation reduces interindividual variability of periodic rebound bursts at steady state

In the intact circuit, the LP neuron generates bursts of action potentials on rebound from regular periodic inhibition by the pyloric pacemaker neurons. To mimic the response of the LP neuron to this periodic input, we hyperpolarized the neuron with 20 −5 nA current pulses, applied periodically with a 1 s on/1 s off protocol. The LP neuron responded to the first few pulses with fewer spikes and longer latencies before the responses stabilized to a steady state (Fig. 5*A–C*).

We analyzed rebound latencies and spike histograms only from the last 10 of the 20 cycles, in the same manner as shown in Figure 4. The steady-state rebound latency was smaller in Proc + CCAP than in control (Fig. 5*E*, Table 5). The only significant difference for spike count parameters was an increase in the total spike count, *a*, in the presence of modulators (Table 5), which is consistent with the results of Figure 4, but there was no modulator-induced change in *t*_1/2_ or *k*. As with our previous measurements, there was no difference for any of the parameters between Proc and Proc + CCAP application. Sham neuromodulator application (see Materials and Methods) did not change the rebound properties (Extended Data Fig. 5-1, example).

Although the application of neuropeptides did not always result in significant differences of the mean values, once again the variability for any of the measured parameters was always lower in the presence of modulators, and this variability increased after wash (Fig. 5*F*). For τ and *t*_1/2_, SD decreased more than the mean, which resulted in smaller CVs in Proc and Proc + CCAP. For *a*, the increase in mean compensated for the increase in SD so that CV decreased. Furthermore, the reduction of the CV of *k* was because of both an increase in mean and a decrease of SD. Raw data for these calculations is available as Extended Data Figure 5-2.

### The influence of modulatory currents on rebound properties in a family of LP model neurons

As shown in the experimental results, peptide modulation is sufficient to reduce the interindividual variability of *f–I* curves and rebound properties at the single-cell level. In the STG, peptide modulators are known to activate an inward current, *I*_MI_, and to modify synaptic properties (Thirumalai et al., 2006; Zhao et al., 2011; Li et al., 2018). Generally, *I*_MI_ increases the excitability of a neuron (Zhao et al., 2010). Given that the experiments were conducted on a synaptically isolated LP neuron, we used modeling to examine whether the activation of *I*_MI_ was sufficient to decrease interindividual variability. To examine the contribution of *I*_MI_ to the reduction of interindividual variability, we built a family of 198 LP model neurons (see Materials and Methods). In these models, the addition of peptides was modeled by increasing the level of ${\bar{g}}_{\mathrm{MI}}$, the maximal conductance of *I*_MI_.

**Figure 6.**
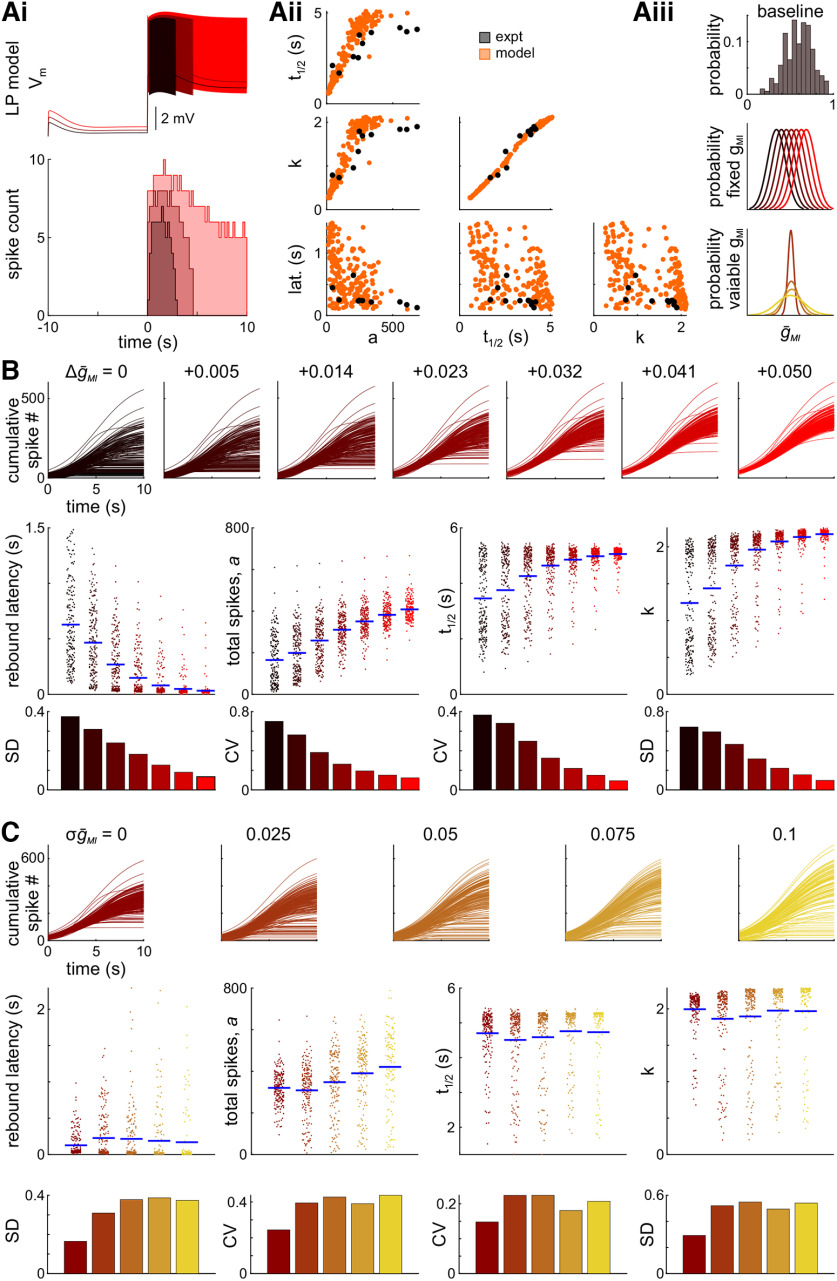
Increasing ${\bar{g}}_{\mathrm{MI}}$ in a family of LP models reduces the variability of rebound parameters. ***A***, A family of 198 LP models was tuned to the rebound statistics from the biological experiments. ***Ai***, Example voltage traces and spike histogram of one of the rebound LP models at different levels of added Δ ${\bar{g}}_{\mathrm{MI}}$. ***Aii***, Paired plots of the rebound parameters from the biological experiments (black) and the family of LP models (orange). ***Aiii***, The baseline ${\bar{g}}_{\mathrm{MI}}$ distribution (top) was shifted by either adding a fixed amount of ${\bar{g}}_{\mathrm{MI}}$ (Δ ${\bar{g}}_{\mathrm{MI}}$, middle) or a variable amount of ${\bar{g}}_{\mathrm{MI}}$ with a fixed mean (σ ${\bar{g}}_{\mathrm{MI}}$, bottom). ***B***, Fits to the cumulative spike histograms (top row), the fit parameters and latency (middle row), and the corresponding measure of variability (bottom row) when adding increasing levels of ${\bar{g}}_{\mathrm{MI}}$ with a fixed distribution (Δ ${\bar{g}}_{\mathrm{MI}}$). Individual dots represent values from an individual LP model, blue bars indicate the mean. An asterisk above a CV bar indicates that the CV for this group is significantly different from the CVs of all other groups. ***C***, Fits to the cumulative spike histograms (top row), the fit parameters and latency (middle row), and the corresponding measure of variability (bottom row) when adding variable levels of ${\bar{g}}_{\mathrm{MI}}$ with a fixed mean (σ ${\bar{g}}_{\mathrm{MI}}$). Individual dots represent values from an individual LP model, blue bars indicate the mean.

We tuned the kinetics of the LP model neurons to allow for rebound spiking as in the biological LP neuron (Fig. 6*Ai*). The models captured the increase and tapering of spike frequency on rebound from hyperpolarization and produced tonic spiking when high levels of *I*_MI_ were activated. The population of LP model neurons was selected for similar rebound properties as measured in the biological data (Fig. 6*Aii*) and had a baseline normal distribution of low levels of ${\bar{g}}_{\mathrm{MI}}$ (Fig. 6*Aiii*, top). To test whether the interindividual variability of rebound decreases in response to modulation, we added fixed amounts of ${\bar{g}}_{\mathrm{MI}}$ (Δ ${\bar{g}}_{\mathrm{MI}}$) to the population of models (Fig. 6*Aiii*, middle). The resulting fits of the cumulative spike histograms are shown in Figure 6*B*.

The metrics of variability were measured as in the biological experiments (see Materials and Methods). We observed a monotonic decrease of variability with increasing amounts of Δ ${\bar{g}}_{\mathrm{MI}}$ added to the population. This suggests that increasing excitability by adding Δ ${\bar{g}}_{\mathrm{MI}}$ is sufficient to decrease the interindividual variability of rebound firing.

The actions of modulators can be inherently variable in magnitude, as supported by reports of variable measurements of *I*_MI_ across individuals and variable receptor expression (Garcia et al., 2015). To address whether variability in the activation of *I*_MI_ would modify the effect seen in the case of adding fixed amounts of Δ ${\bar{g}}_{\mathrm{MI}}$, we allowed Δ ${\bar{g}}_{\mathrm{MI}}$ to be a normal distribution with a moderate mean of Δ ${\bar{g}}_{\mathrm{MI}}$ (0.025) and variances ranging from 0 to 0.1 (σ ${\bar{g}}_{\mathrm{MI}}$; Fig. 6*Aiii*, bottom), resulting in the cumulative histogram fits shown in Figure 6*C*. We found that the effect of increasing σ ${\bar{g}}_{\mathrm{MI}}$ increased the variability of rebound firing compared with adding fixed Δ ${\bar{g}}_{\mathrm{MI}}$. This suggests that the statistics of the ${\bar{g}}_{\mathrm{MI}}$ distribution activated will influence the effect observed on the interindividual variability of rebound firing, where, for a given mean value of ${\bar{g}}_{\mathrm{MI}}$, a narrower distribution will allow for a greater reduction of variability than one with a larger variance.

### Is the reduction of variability only due to an increase in excitability?

**Figure 7.**
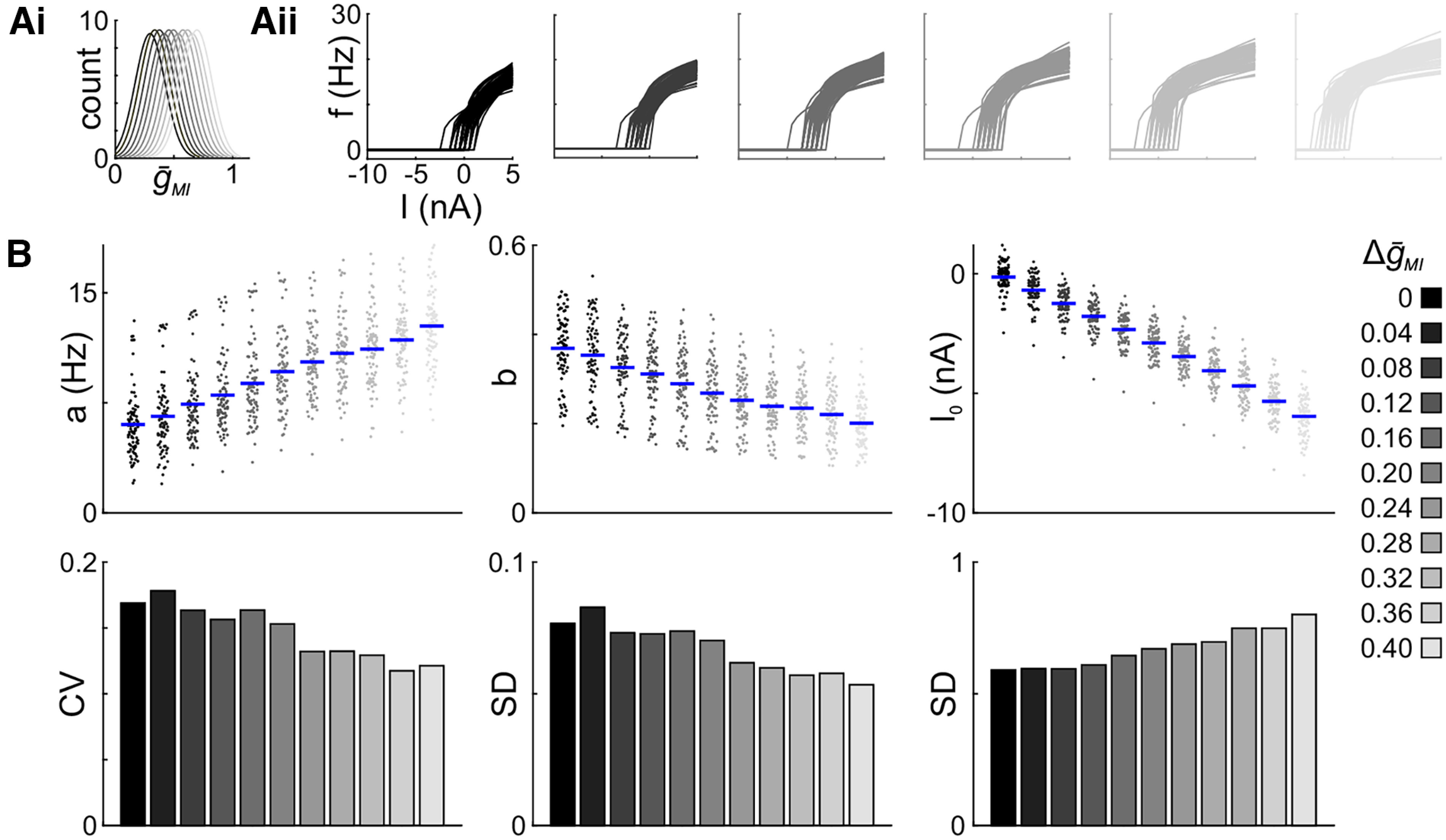
Increasing g_MI_ in a family of LP models reduces the variability of *f–I* parameters. ***A***, Schematic of the right shift of ${\bar{g}}_{\mathrm{MI}}$ to increase excitability (***Ai***) and *f–I* curves of a family of 85 LP models for selected Δ ${\bar{g}}_{\mathrm{MI}}$ values (***Aii***). ***B***, Fit parameters (top row) and corresponding variability measures (bottom row). Dots represent individual experiments; blue bars indicate the mean.

Generally, the increase of ${\bar{g}}_{\mathrm{MI}}$ decreased the variability of rebound parameters in our family of LP model neurons. However, *f–I* relationships capture a different aspect of neuron output than rebound properties. Therefore, we tuned a family of LP model neurons to have *f–I* statistics similar to those observed in the biological experiments and added fixed amounts of ${\bar{g}}_{\mathrm{MI}}$ (Δ ${\bar{g}}_{\mathrm{MI}}$) to the baseline distribution, as we did for the model rebound experiments (Fig. 7*Ai*). We only included models that were reliably producing spikes at all Δ ${\bar{g}}_{\mathrm{MI}}$ values (*n* = 85).

The most obvious change in the *f–I* curves was a left shift with increasing Δ ${\bar{g}}_{\mathrm{MI}}$ (Fig. 7*Aii*). This shift simply reflects a lower threshold for excitability (i.e., a more negative *I*_0_). In addition, the means of scaling factor *a* increased and those of *b* decreased; Fig. 7*B*), indicating, respectively, an increase in the maximum spike rate and an overall reduction in gain. In contrast to the rebound parameters, the *f–I* parameters did not seem to approach a saturating value across the range of Δ ${\bar{g}}_{\mathrm{MI}}$ values. However, we were unable to add larger amounts of Δ ${\bar{g}}_{\mathrm{MI}}$ because models stopped generating spikes due to depolarization block. With increased Δ ${\bar{g}}_{\mathrm{MI}}$, variability decreased only for *a* and *b*, but not for *I*_0_ (Fig. 7*B*). Even so, the reduction in variability was far less than what we observed for the model rebound parameters.

**Figure 8.**
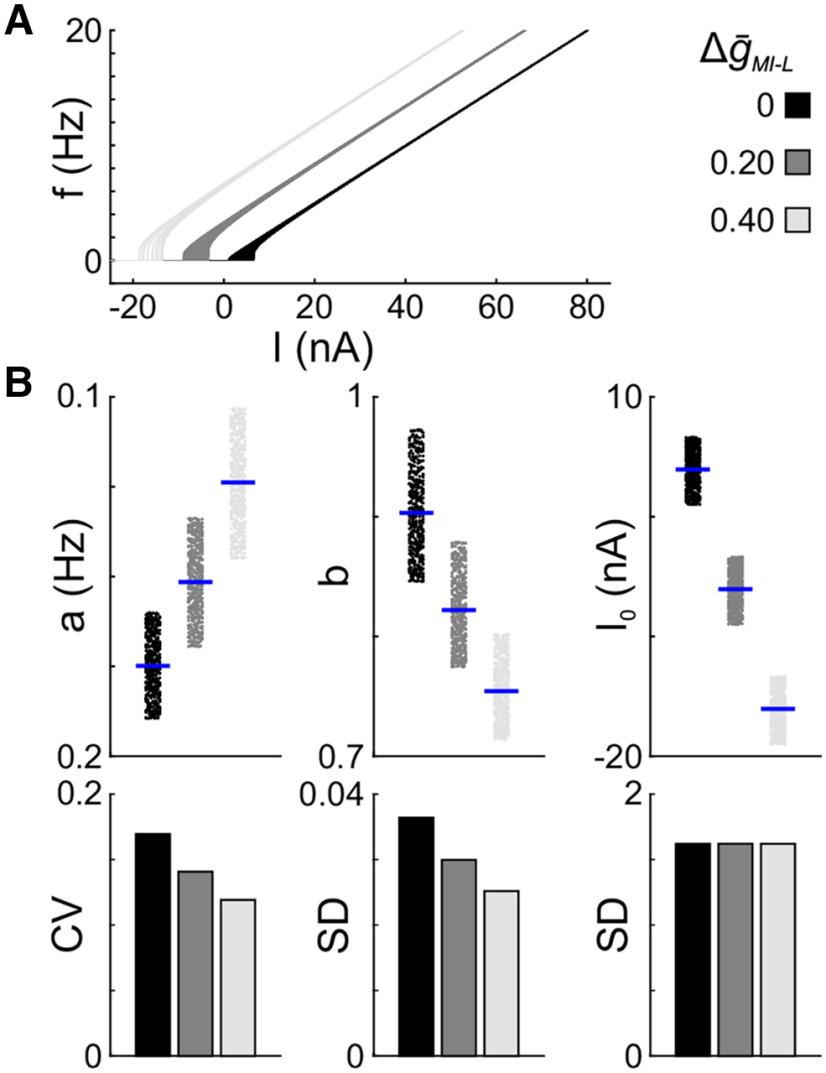
Increasing excitability in a family of LIF models partially reduces the variability of *f–I* parameters. ***A***, *f–I* curves for a family of 500 LIF models at three different Δ ${\bar{g}}_{\mathrm{MI-L}}$ values. As in the LP models, increasing excitability shifts the curves to the left. ***B***, Fit parameters (top row) and corresponding variability measures (bottom row). Dots represent individual experiments; blue bars indicate the mean.

Is the reduction of variability only due to an increase in excitability? To address this question from a first principles perspective, we used a family of LIF models (*n* = 500). Since the frequency for these models does not saturate, we truncated all *f–I* curves at 20 Hz to fit the power function to the same range of frequency values in each condition. The underlying variability within this family of models comes from the variability of the leak conductance. We expected that, like in the other models, increasing excitability in the LIF models (by increasing *I*_MI-L_ levels) would result in a left shift of the *f–I* curves, but the other parameters would not be changed. As expected, *I*_0_ became more negative with increased excitability, and the SD remained constant (Fig. 8). However, the scaling factor (*a*) increased, and the curves became less linear (*b* decreased). The decrease in *b* corresponds to the widening of the base of the *f–I* curves with increasing Δ ${\bar{g}}_{\mathrm{MI-L}}$. The variability of these parameters was reduced in the same way as for the LP model family (Fig. 8). Thus, it appears that the reduction in variability due to increased excitability is a more generic property and is not limited to the LP model neuron.

## Discussion

Neural circuit output can show variability across individual animals (Marder and Taylor, 2011; Wenning et al., 2018; Anwar et al., 2022; Gorur-Shandilya et al., 2022), but some attributes of this output must be constrained to provide biologically meaningful function characteristic for a given circuit state. For example, in the pyloric circuit, oscillation frequency can vary substantially across individuals under control conditions, while the relative timing and duty cycles of different neuron types is maintained (Bucher et al., 2005; Goaillard et al., 2009; Anwar et al., 2022). Such interindividual similarity of circuit output attributes has been carefully documented in central pattern-generating circuits, in which bursting neurons maintain a relatively constant phase in each oscillation cycle despite variations in cycle frequency (Grillner, 2006; Mullins et al., 2011; Zhang et al., 2014; LeGal et al., 2017; Martinez et al., 2019). The mechanisms that constrain aspects of circuit output to give rise to interindividual similarity are not well understood, particularly because ionic currents in identified neurons vary substantially across individuals (Schulz et al., 2006; Khorkova and Golowasch, 2007; Golowasch, 2014; Anwar et al., 2022). It is also not clear to what degree output similarity arises at the level of individual neurons or at the level of the whole circuit. Compounding this puzzle is the fact that circuit output is shaped by the actions of neuromodulators, which can also vary across individuals. Here we show that excitatory neuromodulation can increase the interindividual similarity of response properties in an isolated identified neuron. Further work is required to show whether such reduction of variability translates to the circuit output level, where differentially modulated neurons and synapses increase the complexity of neuromodulator actions (Harris-Warrick and Johnson, 2010; Johnson et al., 2011; Oleisky et al., 2020).

### Variability of modulator-activated currents

Both peptides that we used in this study converge to activate the same ionic current, *I*_MI_, in the LP neuron (Swensen and Marder, 2000, 2001). In the same neuron type, ionic currents, including *I*_MI_, can greatly vary across animals (Schulz et al., 2006; Goaillard et al., 2009; Ransdell et al., 2013a,b), but it is possible that *I*_MI_ has more consistent levels or that it is coordinated with other ionic currents, thereby promoting a similar neural activity. We therefore recorded both unmodulated currents and proctolin-activated currents and found similar levels of variability, indicating that interindividual output similarity is not because of consistent levels of the modulated current or simply because of a reduction in component variability.

Reduction in the variability of response properties must therefore be an emergent property (i.e., it must arise from the way that the currents activated by neuropeptides interact with other currents). Principally, there are two types of current interactions. First, as previous theoretical work has demonstrated, disparate current combinations across individuals can result in similar circuit output and voltage trajectories of individual neurons (Golowasch et al., 2002; Prinz et al., 2004). Indeed, despite substantial interindividual variability, pairs of ionic conductances are often correlated (McAnelly and Zakon, 2000; MacLean et al., 2003; Khorkova and Golowasch, 2007; Schulz et al., 2007; Cao and Oertel, 2011; Amendola et al., 2012; Temporal et al., 2012; Ransdell et al., 2013a; Tran et al., 2019). Such correlations can be independent of activity (MacLean et al., 2005; Schulz et al., 2006) and under neuromodulatory control (Khorkova and Golowasch, 2007; Temporal et al., 2012). Second, ionic currents within a cell have complex and nonlinear interactions through their voltage or Ca^2+^ dependence, as currents both shape and depend on voltage trajectories and Ca^2+^ fluctuations. In fact, *I*_MI_ seems to be partially carried by Ca^2+^ ions and influence internal Ca^2+^ levels (Zhao et al., 2011; Gray et al., 2017; Schneider et al., 2021) and may therefore impact both voltage- and Ca^2+^-dependent currents. Notably, there are substantial differences in how sensitive different neuronal activity attributes are to the variability of different currents (Taylor et al., 2009), suggesting that not all variability has the same functional impact. In addition to their maximal conductances, voltage and Ca^2+^ dependence of ion channels can also covary, and their dependence on neuromodulators can affect this covariation, with potentially substantial consequences for neuronal activity (Amendola et al., 2012).

While we focused on currents that can be easily measured in the soma, the interindividual variability of fast axonal currents in the LP neuron is unknown. However, since currents, such as *I*_MI_, that arise in the neurites can influence neuronal excitability, they can also impact the spiking output that a neuron generates and thus alter activity phases or the number of spikes per burst.

### Potential mechanisms for the reduction of variability by proctolin

Our computational models showed that the activation of *I*_MI_ is sufficient to reduce the interindividual variability of LP activity. Notably, in a family of model LP neurons, adding moderate, but variable, levels of ${\bar{g}}_{\mathrm{MI}}$ increased output similarity compared with low levels of ${\bar{g}}_{\mathrm{MI}}$ with a narrower distribution. Thus, increasing levels of ${\bar{g}}_{\mathrm{MI}}$ can reduce the variability of neural activity even if the ${\bar{g}}_{\mathrm{MI}}$ levels are variable.

Increasing *I*_MI_ increases excitability because *I*_MI_ is a regenerative inward current (Zhao et al., 2010). Our models indicated two effects that contribute to reduced variability. (1) Firing frequency increased more in neurons with an initially low spiking activity, which promoted interindividual similarity. This is reminiscent of the finding that the activation of peptidergic modulatory neurons increases pyloric cycle frequency primarily when the baseline frequency is low (Nusbaum and Marder, 1989; Bartos and Nusbaum, 1997). The effect we describe here is independent of saturation, but rather reflects the larger slope and wider base of the *f–I* curve at low frequencies, as we demonstrated with LIF models that do not saturate (Fig. 8). (2) The other effect was saturation. Neurons have a maximal firing frequency, which is approached when they receive increasing excitation. If maximum firing rates are similar across individuals (i.e., a similar ceiling for firing rates), increasing excitability can reduce variability by reaching this ceiling. Maximum firing rates are constrained by the kinetics of ionic currents, in particular, those that influence the refractory period of the neuron. Similar kinetics would produce similar maximum firing rates. In contrast, highly variable ion channel kinetics across a population may lead to an increase, rather than a decrease, in output variability. Our family of model LP neurons in fact had near-identical ionic current kinetics and were only distinct in the maximal conductances of each current.

Thus, our models show two potential strategies for the system to reduce population variability: allowing for the output attributes to approach a ceiling or shifting these attributes away from low values (i.e., raising the floor).

### Combined application of CCAP and proctolin does not additionally reduce variability

Neuropeptides mostly act through specific G-protein-coupled receptors (Brain and Cox, 2006; Huang and Thathiah, 2015), and in the STG, the subcellular pathways activated by the receptors of excitatory neuropeptides converge downstream to activate *I*_MI_ (Swensen and Marder, 2001; Garcia et al., 2015). In the LP neuron, the combined activation of *I*_MI_ by coapplication of CCAP and proctolin at concentrations >10^−7^ m is simply additive up to saturation (Li et al., 2018). We did not find an additional reduction of variability by coapplication of proctolin and CCAP compared with proctolin alone. Most likely, both 10^−6^ m proctolin and the combination of 5 × 10^−7^ m for each proctolin and CCAP were close to saturating (i.e., activated all *I*_MI_ channels in the LP neuron so that the modulator-mediated reduction in variability was similar in both cases). Interestingly, the combined activation of *I*_MI_ by the coapplication of CCAP and proctolin is sublinear when at least one of them is applied at a lower concentration (Li et al., 2018).

How would neuromodulation at nonsaturating concentrations affect output similarity? If two neuromodulators additively converge to activate a downstream target, but act through independently varying receptors, then their combined actions would result in less variability in the activation levels of that target. This is because adding two independently varying quantities has a smaller variability than either quantity, due to signal averaging. However, as mentioned above, the interaction of peptide modulators in activating *I*_MI_ can be nonlinear (Li et al., 2018), which could result in a more complex interaction in how comodulation influences output variability.

### Potential pitfalls in interpreting variability metrics

To our knowledge, few attempts have been made to quantify interindividual variability or output similarity (Wenning et al., 2018). SD is not dimensionless and therefore impossible to compare, for example, between spike number and current amplitude. Furthermore, SDs of the same unit cannot be compared when the means are very different. In contrast, the CV is dimensionless and scales the SD to the mean, which allows for the direct comparison of variability across different parameters. However, the CV is only valid for data on a ratio scale and therefore could not be used for all parameters. Furthermore, CV can be sensitive to parameters with small means: experimental error introduces some variability in any dataset that does not scale with the parameter of interest. In such cases, dividing SD by a small mean value results in a CV that is most likely an overestimation of the population variability. It is therefore possible that the interindividual variability of small ionic currents in the LP neuron, such as *I*_h_ and *I*_MI_, is influenced by measurement errors and therefore the biological variability is smaller than what we reported.

In our analysis, both the SD and the CV yield only a single value across animals, which makes statistical comparisons difficult. This is different from experiments in which intraindividual variability is compared between different conditions (Arancillo et al., 2015; Boele et al., 2018). In those experiments, a distribution of CVs exists for each condition (one CV per animal per condition), which can be compared with common statistical tests. In contrast, in our experiments, we quantified the interindividual variability with a single value. Unfortunately, available tests to compare the CV equality of single values require data to be normally distributed and only have sufficient power for CV values <0.5 (Sokal and Braumann, 1980; Feltz and Miller, 1996; Krishnamoorthy and Lee, 2014). The parameters and their CVs in our datasets did not always meet those requirements, which is why we refrained from using these tests. However, given that both measures of variability (CV and SD) consistently decreased in the presence of neuromodulators and usually recovered after washing, we are confident that neuromodulators did reduce the interindividual variability in the LP neuron.

## Synthesis

Reviewing Editor: Arianna Maffei, Stony Brook University Decisions are customarily a result of the Reviewing Editor and the peer reviewers coming together and discussing their recommendations until a consensus is reached. When revisions are invited, a fact-based synthesis statement explaining their decision and outlining what is needed to prepare a revision will be listed below. The following reviewer(s) agreed to reveal their identity: Ronald Calabrese.

There is agreement between the reviewers that this is overall a well designed study providing an interesting contribution to the field.

The reviewers raised a few concerns about some of the conclusions and provided suggestions to improve clarity.

Additional analysis as suggested by reviewer #2, would be helpful in further supporting the authors’ main points.

The full comments by the reviewers follow below.

Reviewer #1

This is a systematic study of how neuromodulation affects output variability of individual neuronal firing properties using identified and synaptically isolated LP neurons of the stomatogastric pyloric CPG of crabs. The work is carefully executed and statistical approaches are sound and clearly rationalized and explicated. There is clearly a reduction in output variability (by various rigorous measures comparing CV and SD as is appropriate to the variable) with neuromodulator application observed in experiments and this finding is substantiated in an ensemble modeling approach with realistic and leaky integrate and fire neuronal models. The primary conclusion is that increasing excitability by raising baseline firing accounts for the most to the decrease in variability with modulator action. The writing is very clear and the figure very accessible though complicated. The data is convincing.

Minor Suggestions for Revision

1. I was honestly confused by the Abstract’s ending. "With a family of computational LP-like models, we explored how increased excitability and target variability contribute to output similarity and found two mechanisms. (sic) saturation of the responses and a differential increase in baseline activity. Saturation alone can reduce the interindividual variability only if the population shares a similar ceiling for the responses. In contrast, reduction of variability due to the increase in baseline activity is independent of ceiling effect." I could not follow the arguments in the Discussion about saturation, and indeed I did not see the argument framed as similarity of ceiling effects. I suggest you stick to the main point and not emphasize the saturation angle as I don’t see this explored well.

2. I kept wondering about the separation of the spiking mechanism both in time scale and spatially from the measured currents. Do you think the fast spiking system of the axon is less variable than the slow system of the cell body and primary neurite? As far as I can see axonal fast INa has not been measured in LP neurons (maybe there is a fast IK in the axon area?). You measure currents in the slow system and your output measures are largely the fast system. Can you comment on this in discussion?

3. Lines 474-476: Make clear here in the text that excitability in the LIF models is changed by changing IMI.

Reviewer #2

In this article, the authors investigate the changes in variability of neuronal output associated with neuromodulation in a crustacean central pattern generator motorneuron (LP neuron). After evaluating the variability present at the biophysical level by measuring several ion currents (including the neuropeptide-activated current at the center of the study) in voltage-clamp, the authors use different protocols to characterize the neuronal output, including fI curves and more ecological rhythmic rebound stimuli. The authors analyze the variability of each parameter (by calculating either the standard deviation or the coefficient of variation) under control and neuromodulated conditions in order to determine the changes in variability under neuromodulation. Overall, most electrophysiological parameters display reduced variability under neuromodulation. The authors then use a database modeling approach to demonstrate that this reduction in variability is observed even when the neuromodulator-activated current is highly variable from neuron to neuron. Overall, the study is well performed, both at the electrophysiological and modeling level, the data are analyzed correctly and the manuscript is well written. The authors are also conscious of the limits of the statistical analysis.

MAJOR POINTS

As the authors underline it, comparing variability between conditions is to the least a complex question, because variability is quantified by a single parameter (SD or CV in their case), and thus very little statistical argument can be provided to support the conclusions (is a decrease in CV value really a significant decrease?). Notwithstanding this difficulty, and considering the CV as the main factor that has been used by the authors, it would be important to distinguish between several cases that can lead to a decrease in CV under neuromodulated condition. Since the CV is a ratio between SD and the mean, a decrease in CV can be basically a consequence of three different situations: i) a constant SD associated with an increase in the mean ii) a decrease in SD associated with a constant mean and iii) a decrease in the mean associated with a "stronger" decrease in SD. Since the authors use in some cases the SD instead of the CV (particularly when data are not on a ratio scale), I believe distinguishing between these cases is of the utmost importance. I would suggest that, whenever CV is used to estimate variability, the authors comment on the sources of reduction in variability (is the SD decreased? The mean increased? Do both the SD and the mean change in value?). Perhaps considering the range of values (instead of the CV) could also be of interest for some of the electrophysiological parameters analyzed? I would expect the authors to refine a bit their analysis or discussion of the results to distinguish between the sources of the reduction in variability. This supplementary analysis may also help to better define what may be the biological relevance of these different changes in variability under neuromodulation.

## Author Response

We thank the reviewers for their helpful comments to improve the manuscript. Our responses are highlighted in red.

Reviewer #1

1. I was honestly confused by the Abstract’s ending. "With a family of computational LP-like models, we explored how increased excitability and target variability contribute to output similarity and found two mechanisms. (sic) saturation of the responses and a differential increase in baseline activity. Saturation alone can reduce the interindividual variability only if the population shares a similar ceiling for the responses. In contrast, reduction of variability due to the increase in baseline activity is independent of ceiling effect." I could not follow the arguments in the Discussion about saturation, and indeed I did not see the argument framed as similarity of ceiling effects. I suggest you stick to the main point and not emphasize the saturation angle as I don’t see this explored well.

The period in the abstract should have been a colon; we corrected this in line 14. We reformatted and extended the corresponding section 6.2 in the Discussion to more clearly delineate which data and arguments belong to the discussion about increasing baseline activity or to the discussion about ceiling effects.

2. I kept wondering about the separation of the spiking mechanism both in time scale and spatially from the measured currents. Do you think the fast spiking system of the axon is less variable than the slow system of the cell body and primary neurite? As far as I can see axonal fast INa has not been measured in LP neurons (maybe there is a fast IK in the axon area?). You measure currents in the slow system and your output measures are largely the fast system. Can you comment on this in discussion?

We added this point to the discussion at the end of section 6.1.

3. Lines 474-476: Make clear here in the text that excitability in the LIF models is changed by changing IMI.

Done.

Reviewer #2

As the authors underline it, comparing variability between conditions is to the least a complex question, because variability is quantified by a single parameter (SD or CV in their case), and thus very little statistical argument can be provided to support the conclusions (is a decrease in CV value really a significant decrease?). Notwithstanding this difficulty, and considering the CV as the main factor that has been used by the authors, it would be important to distinguish between several cases that can lead to a decrease in CV under neuromodulated condition. Since the CV is a ratio between SD and the mean, a decrease in CV can be basically a consequence of three different situations: i) a constant SD associated with an increase in the mean ii) a decrease in SD associated with a constant mean and iii) a decrease in the mean associated with a "stronger" decrease in SD. Since the authors use in some cases the SD instead of the CV (particularly when data are not on a ratio scale), I believe distinguishing between these cases is of the utmost importance. I would suggest that, whenever CV is used to estimate variability, the authors comment on the sources of reduction in variability (is the SD decreased? The mean increased? Do both the SD and the mean change in value?). Perhaps considering the range of values (instead of the CV) could also be of interest for some of the electrophysiological parameters analyzed? I would expect the authors to refine a bit their analysis or discussion of the results to distinguish between the sources of the reduction in variability. This supplementary analysis may also help to better define what may be the biological relevance of these different changes in variability under neuromodulation.

All our figures have panels that show, for each parameter, the distribution of data from individual experiments and the mean across individuals, e.g., Fig 2C. For clarification, we added brief descriptions of the main factor (change in SD, change in mean, or change in both) that contributes to the decrease in CV for the biological experiments. Additionally, we now first list the potential mechanisms for decreasing CV in lines 314-317, and include the raw data for Figures 3-5 as Extended Data.

We also realized that contrary to our descriptions in the Methods and Results, we were showing CV instead of SD for the rebound latencies in Figures 4 and 5. The figures have been corrected and show the SDs now.
