# Supplementary figures and images for: Neuromodulation Reduces Interindividual Variability of Neuronal Output
Source: eNeuro. 2022 Aug 5;9(4):ENEURO.0166-22.2022. doi: 10.1523/ENEURO.0166-22.2022 (PMC9361792; doi:10.1523/ENEURO.0166-22.2022)

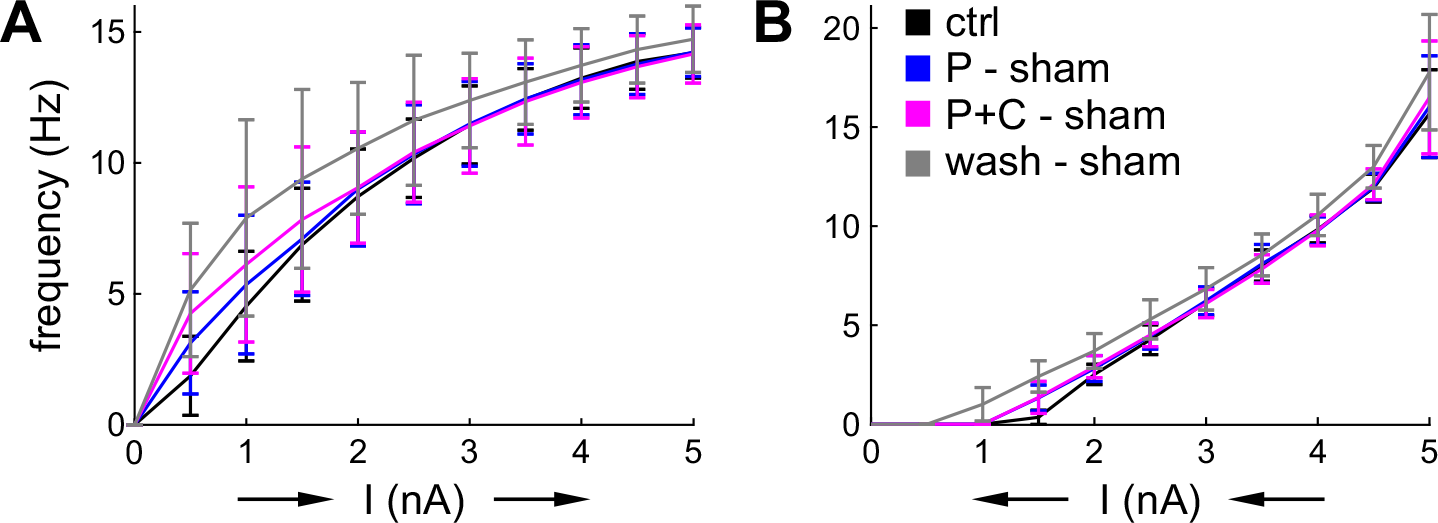

Supplement: Figure 3-1 — f–I relationships with sham applications of neuromodulators. One example experiment. A, Increasing current application (indicated by arrows). B, Decreasing current application (indicated by arrows). Download Figure 3-1, TIF file. [file enu-eN-NWR-0166-22-s04.tif]

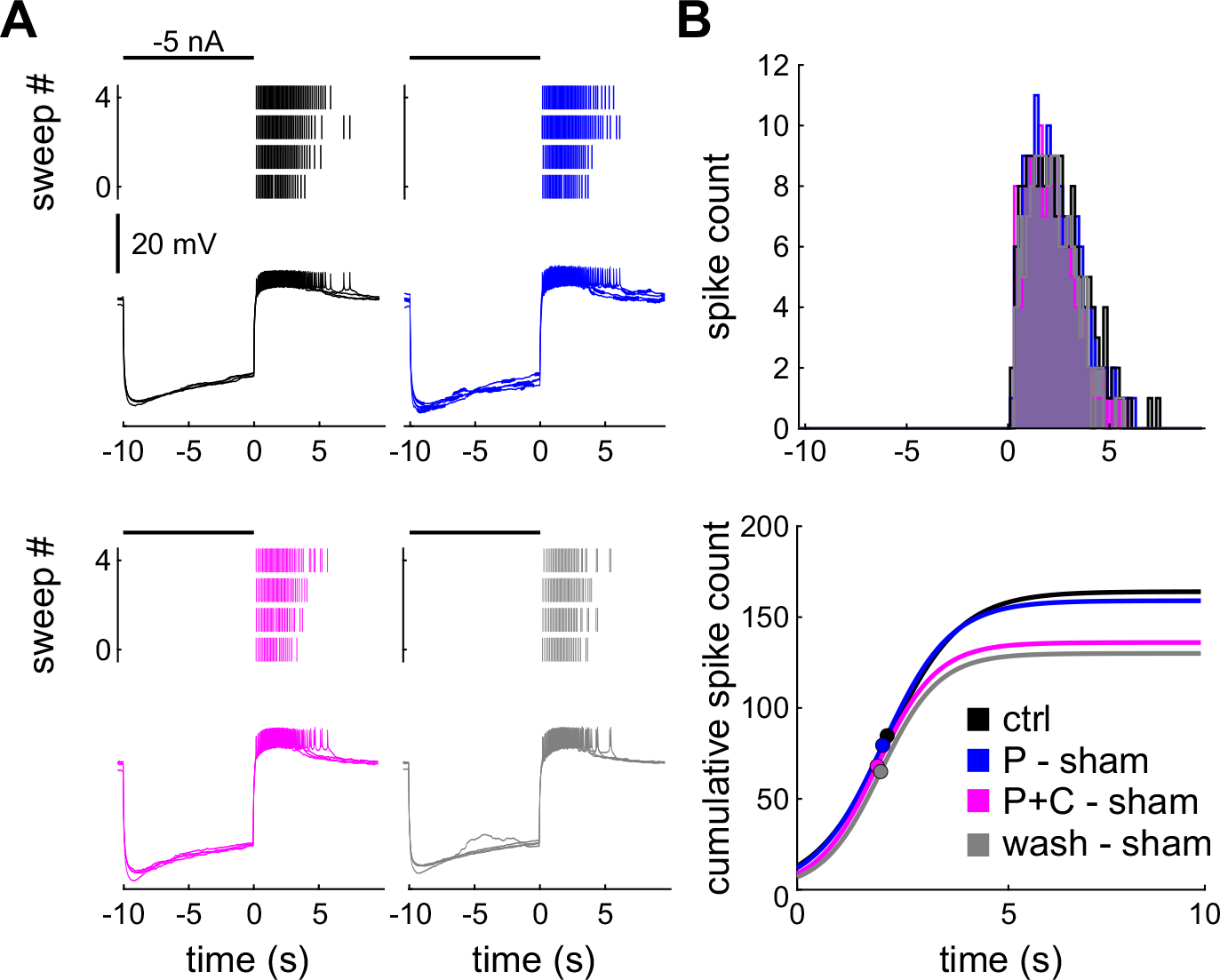

Supplement: Figure 4-1 — Rebound with sham applications of neuromodulators. One example experiment. A, Spike raster and corresponding intracellular recordings for all five sweeps. B, Spike histogram and sigmoid fit to the cumulative spike histogram. Dots indicate sigmoid midpoint. Download Figure 4-1, TIF file. [file enu-eN-NWR-0166-22-s05.tif]

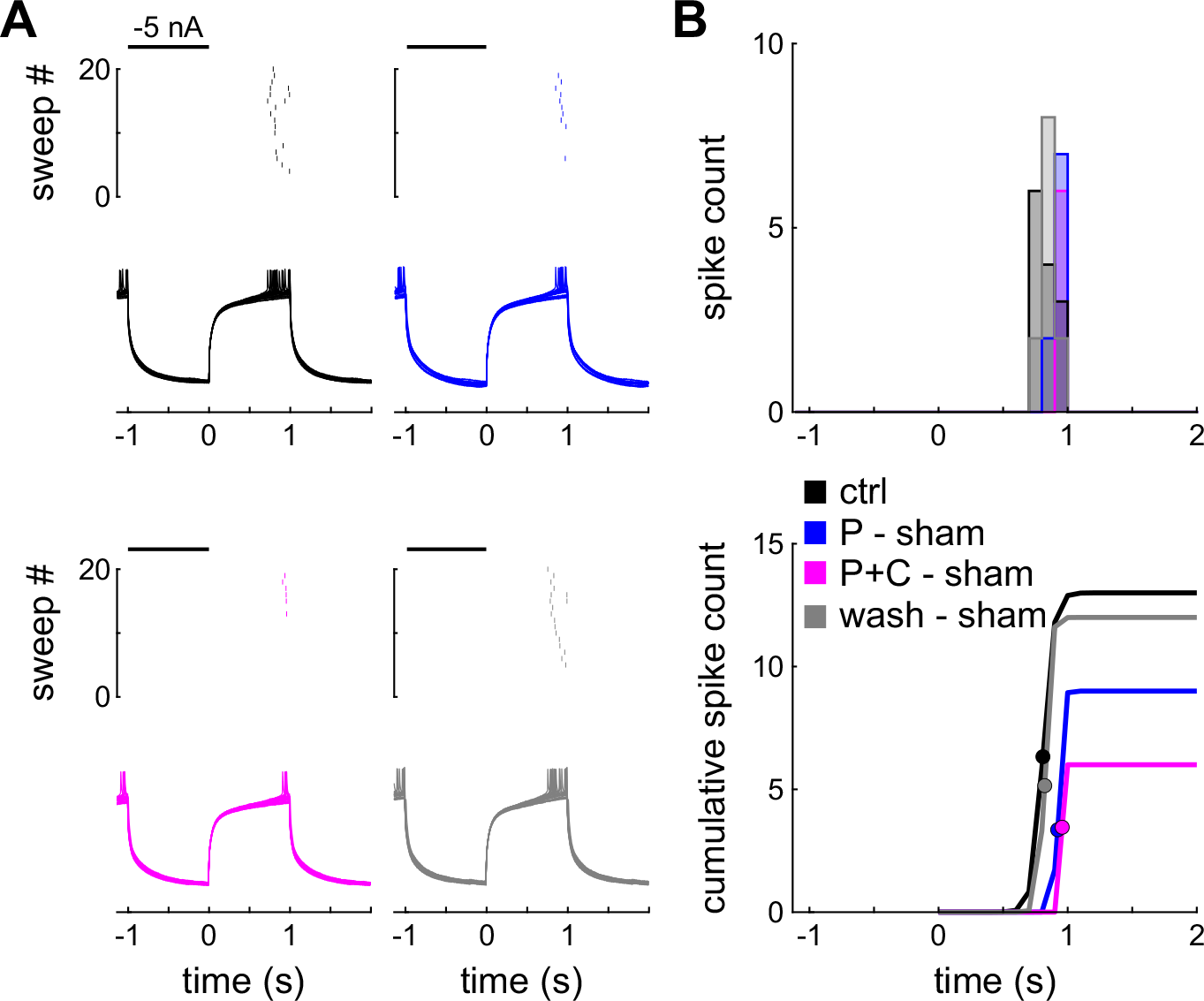

Supplement: Figure 5-1 — Periodic rebound with sham applications of neuromodulators. One example experiment. A, Spike raster and corresponding intracellular recordings for all 20 sweeps. B, Spike histogram and sigmoid fit to the cumulative spike histogram. Dots indicate sigmoid midpoint. Download Figure 5-1, TIF file. [file enu-eN-NWR-0166-22-s06.tif]
